# Downregulation of miR‐326 and its host gene β‐arrestin1 induces pro‐survival activity of E2F1 and promotes medulloblastoma growth

**DOI:** 10.1002/1878-0261.12800

**Published:** 2020-12-31

**Authors:** Evelina Miele, Agnese Po, Angela Mastronuzzi, Andrea Carai, Zein Mersini Besharat, Natalia Pediconi, Luana Abballe, Giuseppina Catanzaro, Claudia Sabato, Enrico De Smaele, Gianluca Canettieri, Lucia Di Marcotullio, Alessandra Vacca, Antonello Mai, Massimo Levrero, Stefan M. Pfister, Marcel Kool, Felice Giangaspero, Franco Locatelli, Elisabetta Ferretti

**Affiliations:** ^1^ Department of Pediatric Hematology and Oncology, Cell and Gene Therapy Bambino Gesù Children's Hospital, IRCCS Rome Italy; ^2^ Department of Molecular Medicine Sapienza University Rome Italy; ^3^ Neurosurgery Unit, Department of Neurological and Psychiatric Sciences Bambino Gesù Children's Hospital, IRCCS Rome Italy; ^4^ Department of Experimental Medicine Sapienza University Rome Italy; ^5^ Center for Life NanoScience@Sapienza Istituto Italiano di Tecnologia Rome Italy; ^6^ Department of Chemistry and Technologies of Drugs Sapienza University of Rome Italy; ^7^ Cancer Research Center of Lyon (CRCL), UMR Inserm 1052 CNRS 5286 Mixte CLB Université de Lyon 1 (UCBL1) France; ^8^ Department of Internal Medicine and Medical Specialties Sapienza University Rome Italy; ^9^ Division of Pediatric Neurooncology German Cancer Research Center DKFZ Heidelberg Germany; ^10^ Department of Pediatric Oncology, Hematology and Immunology University Hospital Heidelberg Germany; ^11^ Hopp Children's Cancer Center Heidelberg (KiTZ) Heidelberg Germany; ^12^ Department of Radiological, Oncological and Pathological Science Sapienza University Rome Italy; ^13^ IRCCS Neuromed Pozzilli Italy; ^14^ Department of Maternal Infantile and Urological Sciences Sapienza University Rome Italy

**Keywords:** ARRB1, E2F1, EZH2, medulloblastoma, miR‐326

## Abstract

Persistent mortality rates of medulloblastoma (MB) and severe side effects of the current therapies require the definition of the molecular mechanisms that contribute to tumor progression. Using cultured MB cancer stem cells and xenograft tumors generated in mice, we show that low expression of *miR‐326* and its host gene *β‐arrestin1 (ARRB1)* promotes tumor growth enhancing the E2F1 pro‐survival function. Our models revealed that miR‐326 and ARRB1 are controlled by a bivalent domain, since the H3K27me3 repressive mark is found at their regulatory region together with the activation‐associated H3K4me3 mark. High levels of EZH2, a feature of MB, are responsible for the presence of H3K27me3. Ectopic expression of miR‐326 and ARRB1 provides hints into how their low levels regulate E2F1 activity. MiR‐326 targets E2F1 mRNA, thereby reducing its protein levels; ARRB1, triggering E2F1 acetylation, reverses its function into pro‐apoptotic activity. Similar to miR‐326 and ARRB1 overexpression, we also show that EZH2 inhibition restores miR‐326/ARRB1 expression, limiting E2F1 pro‐proliferative activity. Our results reveal a new regulatory molecular axis critical for MB progression.

## Graphical abstract

Medulloblastoma (MB) is a malignant pediatric brain tumor, and we reveal a new regulatory molecular axis critical for MB progression. Low expression of *miR‐326* and its host gene *β‐arrestin1 (ARRB1)* promotes tumor growth enhancing the E2F1 pro‐survival function. High levels of EZH2 are responsible for the presence of H3K27me3 that controls miR‐326 and ARRB1 through a bivalent domain. Restoration of miR‐326 and re‐expression of ARRB1 reverse E2F1 function into pro‐apoptotic activity.

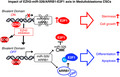

**Abbreviations**
ARRB1: β‐arrestin1
BTC: bulk tumor cell
CSCs: cancer stem cells
EZH2: enhancer of zeste homolog 2
GCP: granule cell progenitors
MB: medulloblastoma
OFC: oncosphere‐forming cell

## 1 Introduction

Brain tumors are an important cause of cancer‐related morbidity and mortality in children, and medulloblastoma (MB) is the most common pediatric malignant brain tumor. High‐throughput analyses have identified at least four subgroups of MB—WNT (Wingless)‐driven MBs, SHH (Sonic hedgehog)‐driven MBs, G3 (Group 3) MBs, G4 (Group 4) MBs [1, 2, 3, 4, 5]. Therapeutic approaches consist mainly of maximally safe surgical resection, high‐dose cytotoxic chemotherapy and, for patients over the age of three, craniospinal irradiation. Although these methods have substantially improved survival, they are frequently associated with severe long‐term adverse effects, and approximately, one‐third of patients still die from the disease [6, 7]. A subpopulation of cancer cells with stem‐like features, referred to as cancer stem cells (CSCs), has been derived, identified, and characterized by us and other research groups in MBs [8, 9] and are considered to be the ultimate source of cancer cells, leading to cancer growth [10, 11].

The urgent need to identify novel potential therapeutic strategies has stimulated interest in understanding the mechanisms sustaining MB growth and maintenance. We previously identified a subset of microRNAs with remarkably low expression levels in MBs [12, 13]. These microRNAs were expressed at low levels in cerebellar granule cell progenitors (GCPs), the proliferating and undifferentiated cells of the developing cerebellum, described as cell of origin of certain MBs [14]. Differentiation of GCPs into cerebellar granule cells was associated with upregulation of these microRNAs, which contributed to this critical transition by inhibiting proliferative signaling [13]. Among these, miR‐326 belongs to a class of neuronal microRNAs that act as translational regulators of neuronal gene expression, with high expression in the cortex and cerebellum [15]. Notably, miR‐326 was shown to have an onco‐suppressive role [16, 17, 18, 19, 20] and low levels of miR‐326 have been reported in brain tumors of glial origin [21, 22, 23].

The gene encoding miR‐326 is embedded within the first intron of the *β‐arrestin1* gene (*ARRB1*) on human chromosome 11q, and its expression is co‐regulated with that of its host via shared promoter sequences [24].

ARRB1, as miR‐326, is involved in neuronal differentiation, where its upregulated expression in cerebellar GCPs and in neural stem cells halts proliferation and induces growth arrest [25, 26]. Notably, under‐expression of ARRB1 has been documented in brain tumors [23, 27, 28, 29, 30, 31, 32].

These reports, combined with the results of our studies, prompted us to further investigate the potential role of miR‐326 and ARRB1 expression patterns and their functional implications in MBs.

## 2 Materials and methods

Unless otherwise stated, all commercial products were used according to manufacturers' instructions.

### 2.1 Patients and MB samples

Medulloblastoma tumor specimens from two independent cohorts were obtained with the written informed consent of patients or their legal representatives, and the investigation was approved by the Institutional Review Board of the contributing centers (Prot. N. 21LB; Study Number 730/2013 Bambino Gesù Hospital) in accordance with the Helsinki declaration of 1964 and its later amendments. Cohort 1 comprised of 84 patients (Table S1) treated at the Bambino Gesù Children's Hospital (Rome, Italy): 34 have already been described elsewhere [12, 13], while 50 others who underwent surgery between 1 January 2013 and 20 January 2016 have not been described yet. Cohort 2 included 437 patients recruited by the German Cancer Research Center (Heidelberg), 62 of whom have been described elsewhere [1].

Formalin‐fixed paraffin‐embedded (FFPE) samples of each tumor were re‐examined by a single neuropathologist (F.G.), who confirmed the original diagnosis or revised it to reflect international consensus guidelines. Additional tumor samples from cohort 1 were collected from each MB. One (~ 0.5 cm^3^) was snap‐frozen in liquid nitrogen, stored at −80 °C, and used for RNA extraction; the second was used to isolate MB CSCs, as described below.

### 2.2 Control RNA samples

RNAs from normal human cerebella (10 samples from adults aged 25–70 years) were purchased from Biocat (Heidelberg, Germany), Ambion (Applied Biosystems, Foster City, CA, USA), and BD Biosciences (San Jose, CA, USA).

### 2.3 RNA isolation and expression analyses

Total RNA was purified and reverse‐transcribed as previously described [13]. Quantitative RT‐PCR (qRT‐PCR) analysis was performed with the ViiA7 Sequence Detection System (Thermo Fisher Scientific, Waltham, MA, USA) and best‐coverage TaqMan gene expression assays specific for each mRNA analyzed. MB subgroup classification was performed by qRT‐PCR using TaqMan probes, as described elsewhere [33, 34]. One microgram of RNA was reverse‐transcribed using the High‐capacity cDNA Reverse Transcription Kit (Thermo Fisher Scientific). Each amplification was performed in triplicate, and the average of three threshold cycles was used to calculate transcript abundance. Transcript quantification was expressed in arbitrary units as the ratio of the sample quantity to the calibrator or to the mean values of control samples. All values were normalized to four endogenous gene controls: GAPDH, β‐actin, β2‐microglobulin, and HPRT. Mature miR‐326 levels were assessed as previously described [12].

For cohort 2 MBs, mRNA expression Array analysis was performed by Affymetrix Human U133 Plus2.0 arrays (Thermo Fisher Scientific) and microRNA analysis was performed by miR‐seq.

### 2.4 Cell lines

Medulloblastoma cells (CHLA, DAOY, D283, and D341) and HEK293 cells were purchased from and authenticated by the American Type Culture Collection (ATCC, Manassas, VA, USA).

Patient‐derived MB stem cell‐like cell lines 1–6 (MB CSC_1–6_) were derived from MB tissues freshly resected from pediatric patients among the cohort 1 and MB stem‐like cells were derived from D283 cancer cells (D283 CSC). As previously described [8], bulk tumor cells (BTCs) were grown in stem‐cell medium (SCM) consisting of DMEM/F12 supplemented with 0.6% glucose, 25 mg·mL^−1^ insulin, 60 mg·mL^−1^ *N*‐acetyl‐l‐cysteine, 2 mg·mL^−1^ heparin, 20 ng·mL^−1^ EGF, 20 ng·mL^−1^ bFGF (Peprotech, Rocky Hill, NJ, USA), 1× penicillin‐streptomycin, and B27 supplement without vitamin A. The oncospheres formed under these conditions were considered MB CSC‐enriched cultures and used for subsequent experiments (Fig. S2).

The frequencies of repopulating cells in primary MB BTCs and MB CSC‐enriched cultures were compared using limited dilution assays, as follows:

#### Primary and secondary sphere formation assays

Bulk tumor cells were centrifuged at 500 ***g*** and seeded into 96‐well plates at densities ranging from 1 to 500 cells per well. After 3–21 days, spheroid colonies (primary oncospheres) were identified. For secondary sphere formation assays, primary oncospheres were dissociated nonenzymatically [Cell Dissociation Solution Non‐enzymatic (C5914); Sigma‐Aldrich, St. Louis, MO, USA) and then mechanically using a fire‐polished Pasteur pipette. Cells thus obtained were plated into 96‐well plates at densities of 1–100 cells/well and clones counted 15 days later. For each plating density, the proportion of wells containing no oncospheres (negative wells) was recorded and plotted against the number of cells plated per well. The fraction of negative wells vs. cell dilution was graphed and fitted with a linear regression to estimate stem cell frequency, as previously described [35]. Assuming that a single stem cell gives rise to one sphere, the proportion of negative wells can be defined by the zero point (F0) of the Poisson distribution (F0 = e^−^ *^x^*, where *x* is the mean number of cells per well). The dilution at which one expects to have one stem cell per well can be identified by the point at which the line‐of‐best‐fit crosses 0.37 (when *x* = 1, F0 = e^−1^ = 0.37) [35].

MB CSC subcultures were obtained by mechanically dissociating MB oncosphere and re‐seeding the cells at a density of 50 000 viable cells·mL^−1^. None of the MB CSCs used in experiments had undergone more than seven passages. Stemness markers (NANOG and CD133) expression profiles were determined for each MB CSC line, as previously reported [8, 36].

#### Assessment of pluripotency

Medulloblastoma oncospheres were mechanically dissociated and the cells plated into d‐poly‐lysine‐coated dishes in *differentiation medium* (DFM) consisting of DMEM/F12 with N2 supplement and 2 mg·mL^−1^ heparin, 0.6% glucose, 60 mg·mL^−1^ *N*‐acetyl‐l‐cysteine, and 1% FBS. Cells were harvested after 48 h unless otherwise specified in figures. The oncosphere ability for multilineage was confirmed by the expression of neuronal (βIII tubulin) and astrocyte markers (GFAP), Fig. S2D,E.

Granule cell progenitors (GCPs) were isolated from postnatal day 4 mice as described in [13, 37] and treated with Sonic Hedgehog ligand (SHH).

### 2.5 Drugs

GSK126 was purchased from ActiveBiochem (Kowloon Bay, Kowloon, Hong Kong) and cells were treated with 5 µm for 48 h. MC3629 was synthesized as previously described [38].

### 2.6 Western blotting

Cells were lysed in Tris/HCl pH 7.6 50 mm, deoxycholic acid sodium salt 0.5%, NaCl 140 mm, NP‐40 1%, EDTA 5 mm, NaF 100 mm, Na pyrophosphate 2 mm, and protease inhibitors. Lysates were separated on 6% or 8% acrylamide gel and immunoblotted using standard procedures. The list of antibodies is included in Appendix S1. HRP‐conjugated secondary antibodies (Santa Cruz Biotechnology, Dallas, TX, USA) were used in combination with enhanced chemiluminescence (ECL, Pierce, Thermo Fisher Scientific). Quantification was performed using imagej version 1.53 [39] as described in the imagej documentation.

### 2.7 Immunoprecipitation assays

HEK293 cells and GCPs were lysed in NET buffer [50 mm Tris/HCl (pH 7.5), 150 mm NaCl, 0.1% Nonidet P‐40, 1 mm EDTA (pH 8), 0.25% gelatin]. One milligram of HEK293 and GCPs extracts was immune‐precipitated overnight on a rocking platform at 4 °C with the indicated antibodies (2 μg) or IgG relevant control and incubated with protein A or protein G Plus (Pierce, Thermo Fisher Scientific) for 2 h at 4 °C. The protein G–antigen–antibody complexes were washed three times with NET buffer, resuspended with LDL sample buffer and heated at 70 °C for 10 min. Samples were analyzed by electrophoresis with Tris‐acetate or Bis‐Tris mini‐gels. The list of antibodies is included in Appendix S1.

### 2.8 *In situ* hybridization

All reagents used before probe hybridization were prepared using diethyl pyrocarbonate‐treated water (Sigma‐Aldrich, D5758) to prevent ribonuclease contamination. Cells were fixed with 4% paraformaldehyde (PFA) for 10 min at room temperature and incubated with 10 μg·mL^−1^ Proteinase K (Sigma‐Aldrich, P2308) (2 min at 37 °C). To increase signal : background ratios, cells were incubated with constant stirring for 10 min at room temperature with 1.2% triethanolamine (Sigma‐Aldrich, 90279), 0.0018 N HCl, and 0.25% acetic anhydride (Sigma‐Aldrich, A6404). Prehybridization was performed using 50% formamide (Sigma‐Aldrich F9037), 5× saline‐sodium citrate (SSC) buffer (FLUKA cat. S6639‐1L), 0.1% Tween‐20, 9.2 mm Citric Acid (Sigma‐Aldrich C1909), 50 μg·mL^−1^ heparin (Sigma H4784), and 500 μg·mL^−1^ Yeast RNA (Sigma‐Aldrich R6750) for 3 h at 62 °C. After 5 min denaturation at 85 °C, the hybridization probe [double DIGlabeled, Exiqon (now Qiagen), Aarhus, Denmark] was cooled on ice, added to cells (at 25 nm), and incubated overnight at 62 °C. Samples were incubated with 0.1× saline‐sodium citrate (SSC) buffer for 3 h at 67 °C and washed in Buffer W (0.1 m Tris/HCl pH 75 and 0.15 m NaCl). Nonspecific antibody binding was performed with 0.5% Blocking Reagent (Roche 11096176001, Basel, Switzerland), 5% Sheep Serum (Sigma‐Aldrich S3772), in 50 mm Tris/HCl pH 7.5, 5 mm EDTA for 2 h at room temperature in a humidified chamber. Fluorescein‐conjugated anti‐DIG was incubated in blocking buffer overnight at 4 °C. Samples were washed with Buffer W, and coverslips were mounted using anti‐fade medium (DAKO S3023, Agilent Technologies, Santa Clara, CA, USA). Fluorescence was visualized, and images were acquired with an Axio Observer Z1 microscope using ApoTome technology and axiovision Digital Image Processing Software (Carl Zeiss AG, Oberkochen, Germany).

### 2.9 Immunofluorescence

MB CSCs were cultured in Lab‐Tek chamber slides fixed in 4% paraformaldehyde for 20 min at room temperature, permeabilized with 0.1% Triton X‐100 cells, incubated in blocking buffer (PBS with 1% BSA) for 30 min, and then with primary antibody overnight in blocking solution at 4 °C. Cell was stained with mouse monoclonal antibodies against βIII tubulin (MAB 1637; Merck, Darmstadt, Germany) and GFAP (MAB360). Nuclei were counterstained with Hoechst (H6024 Sigma‐Aldrich). At least 300 nuclei were counted in triplicate and the number of βIII tubulin‐ or GFAP‐positive cells was recorded.

### 2.10 Overexpression experiments

MicroRNA miR‐326 vector and its negative control were purchased from GeneCopoeia, Rockville, MD, USA (MmiR3333‐MR01); the ARRB1 vector was from Addgene, Watertown, MA, USA (plasmid #14693). MYC‐tagged E2F1 was cloned as previously described [40]. K. Helin kindly provided the mutant *E2F1* expression vectors. The Amaxa Nucleofector (Lonza, Basel, Switzerland) was used to transfect MB CSCs, while Lipofectamine 2000 (Invitrogen, Carlsbad, CA, USA) was used for HEK293 transfections.

### 2.11 Knockdown experiments

For lentiviral transduction of specific anti‐EZH2, short‐hairpin lentiviral particles were purchased from Sigma‐Aldrich: MISSION shRNA‐non target control Transduction Particles (SCH002V) and three Lenti shEZH2: MISSION shRNA EZH2 Lentiviral Clone TRCN0000040074, TRCN0000040077, and TRCN0000010475 (SHCLNV). Clone TRCN0000010475 (SHCLNV) was used because it produced the most efficient knockdown with the fewest off‐target effects.

### 2.12 Assays of cell proliferation, oncosphere formation, and apoptosis

For all assays, cells were seeded in 12‐well plates at a density of 1 × 10^4^ cells/well. Proliferation was evaluated with a BrdU‐labeling assay (Roche Applied Sciences, Penzberg, Germany) or an MTT‐based proliferation assay system (Promega, Madison, WI, USA). Oncosphere‐forming cell (OFC) frequency was assessed by counting the number of oncospheres in each well normalized on plated cells. The OFC frequency in treated cells was expressed as a percentage of that observed under control conditions (%OFC frequency). Apoptosis was detected by terminal deoxynucleotidyl transferase‐mediated UTP nick end labeling (TUNEL) assay performed with the in Situ Cell Death Detection Kit, Fluorescein (Roche Applied Sciences, cat. no. 1684795).

### 2.13 Immunohistochemistry

All experiments were performed on 3‐µm FFPE sections. Sections of normal adult cerebellum (*n* = 3) (BioChain, Newark, CA, USA) and human MBs (cohort 1) were stained with anti‐β‐arrestin1 K‐16 antibody (sc‐8182; Santa Cruz Biotechnology; 1 : 200) and anti‐E2F1 C‐20 (sc‐193; Santa Cruz Biotechnology). Sections of MB CSC xenografts were stained with hematoxylin and eosin (H&E) and anti‐Ki67 (M7240, Dako; 1 : 1000). In detail, deparaffinized and rehydrated sections were quenched with hydrogen peroxide and methanol. Epitope unmasking was achieved with proteinase K (Dako) for β‐arrestin1 or with microwaving in citrate buffer for Ki67 and E2F1. Sections were blocked with Superblock (Scytek Laboratories, Logan, UT, USA) for 5 min and incubated with primary antibodies. Cells were then incubated at RT with biotinylated horse anti‐goat or goat anti‐polyvalent secondary antibody (Scytek Laboratories) (30 min) and streptavidin‐horseradish peroxidase (15 min). The chromogenic reaction was developed with 3,3′‐diaminobenzidine tetrahydrochloride (DAB) solution. The nuclei were counterstained with hematoxylin. Negative control staining without primary antibodies was performed as well. A single observer counted and recorded the percentage of Ki67‐positive (proliferating) cells in each section.

### 2.14 Chromatin immunoprecipitation

Chromatin extracts were analyzed with the MAGnify Chromatin Immunoprecipitation System kit (Invitrogen). Sheared chromatin was immunoprecipitated with 5 µg of anti‐β‐arrestin1 (Clone 10, cat. 610550, BD Biosciences, San Jose, CA, USA) or anti‐HA.11 (Clone 16B12, Covance, Princeton, NJ, USA). Normal mouse IgG furnished with the kit was used as the negative control. Eluted DNA was amplified by qPCR using EpiTect ChIP qPCR Assay (Qiagen, Hilden, Germany) for the following genes: Human *CDC25A*: GPH1022957(‐)01A; Human *TP73* GPM1030084(‐)01A; Human *CASP3*: GPH1024002(‐)01A; Human *CASP7*: GPH1001897(‐)01A. *ACTIN* and *GAPDH* genes were used as controls. Data are presented as input percentage enrichment over background.

Chromatin immunoprecipitation for bivalent domain assessment was performed using the following antibodies: rabbit polyclonal Trimethyl‐Histone H3 (Lys 27) (Merck), rabbit polyclonal Histone H3 (trimethyl K4) (Abcam), and rabbit polyclonal EZH2 (Active Motif, Belgium, Germany) were carried on as previously described [8]. DNA was amplified by PCR with primers on the miR‐326 and ARRB1, regulating region and retrieved by Rulai database (rulai.cshl.edu/TRED/). A standard curve for each primer pair was generated with different dilutions of Input chromatin, and used to quantify the immunoprecipitates. Not related region (NRE) in exon 5 and 11 of the ARRB1 gene or the ACTIN gene were used as control. Oligonucleotides used for PCR amplification are reported in Table S2.

### 2.15 Mice

Adult female NOD‐SCID IL2Rgammanull mice were purchased from Charles River Laboratories, Wilmington, MA, USA and maintained in the Animal Facility at Sapienza University of Rome. All procedures were approved by the University's Ethics Committee for Animal Experimentation (Prot. N 752/2017‐PR) and carried out in accordance with the Guidelines for Animal Care and Use of the National Institutes of Health.

### 2.16 Medulloblastoma xenografts

Orthotopic MB xenografts (XTs) were generated in adult female NOD‐SCID IL2Rgammanull mice via infusion of CSCs derived from primary MB CSC_1–6_ and D283 cells (D283 CSC). When indicated, MB CSCs were transfected with miR‐326 and ARRB1 plasmids or empty vector for 48 h prior to implantation. Additionally, where indicated, MB CSCs were infected with shRNA‐non target control (Mock) or Lenti shEZH2 transduction particles [Clone TRCN0000010475 (SHCLNV) Sigma‐Aldrich]. Where indicated, XTs were treated with EZH2 inhibitor (MC3629), described in [38], or vehicle [10% (2‐Hydroxypropyl)‐β‐cyclodextrin + 1% DMSO (Sigma)], for 21 days, starting from day 7.

Mice were anesthetized by intraperitoneal injection of ketamine (10 mg·kg^−1^) and xylazine (100 mg·kg^−1^). The posterior cranial region was shaved and placed in a stereotaxic head frame. For *in vivo* limiting dilution analyses, MB CSCs were stereotaxically implanted at different cell concentrations (2 × 10^5^, 5 × 10^4^ and 5 × 10^3^ per 3 µL). MB cells (*n* = 200 000) were resuspended in 5 µL of sterile PBS and infused into the cerebellum (rate: 1 µL·min^−1^) using the following stereotactic coordinates [41]: 6.6 mm posterior to the bregma; 1 mm lateral to the midline; and 2 mm ventral from the surface of the skull. After injection, the cannula was kept in place for about 5 min for equilibration of intracranial pressures. The skin was closed over the cranioplasty assembly with metallic clips. In all experiments, eight animals were used for every experimental point.

Mice were sacrificed at the onset of neurological symptoms and/or 28 days after implantation (D283 CSCs) and/or 90 days after implantation (MB CSCs). Brains were removed, fixed in 4% formaldehyde in 0.1 m phosphate buffer (pH 7.2), and paraffin embedded. Brain tumor volume was assessed as follows: serial coronal sections (2 µm) were cut from the beginning of the mesencephalon to the end of cerebellum. The analysis was performed on 20 sections of 2 µm, sampled every 40 µm on the horizontal plane of the cerebellum, in which the cerebellum was identified and outlined at 2.5× magnification. Every 40 µm of brain slice, H&E staining was performed. The tumor area in every slice was evaluated as previously described [42]. Sections were scanned using Aperio Imagescope (Leica Biosystems, Wetzlar, Germany). Images of the whole sections were taken at 1× (upper picture for each panel, scale bar 3 mm) and the detail of the tumor mass at 5× (below each whole section, scale bar 500 µm), as shown in Fig. S8C and Fig. S10. Xenograft sections were stained for apoptotic cells, using the In Situ Cell Death Detection Kit, POD (Roche) with peroxidase detection of TUNEL labeling, according to manufacturer's instructions. Representative images of each sample/stain combination were captured (at 40× original magnification) and subjected to light microscopy with a Jenoptik ProgRes Speed XT Core5 Microscope Camera and progres capture pro 2.8 software (Jenoptik, Jena, Germany). A single observer scored each section for apoptotic index (percentage of TUNEL positive cells). Xenograft sections were also stained with anti‐Ki67 (RRID: AB2250503) detailed in Immunohistochemistry Methods section.

### 2.17 Statistical analysis

All analyses were performed with prism Software, Version 6.0 (GraphPad Prism, San Diego, CA, USA). Differences were analyzed with ordinary one‐way ANOVA followed by Dunnett's or Tukey multiple comparisons test (for evaluation of more than two groups/samples) or two‐way ANOVA followed by Bonferroni's multiple comparisons test (for evaluation of more than two groups/samples in different conditions) or Wilcoxon signed‐rank test for paired data (for one sample in two different conditions) and Mann–Whitney or Student's *t*‐test for independent samples (for two different groups/samples). Adjusted *P* values < 0.05 were considered statistically significant. Results are expressed as means ± SD from an appropriate number of experiments (as indicated in Figure Legends).

## 3 Results

### 3.1 Expression levels of miR‐326 and ARRB1 in MBs and MB CSCs

**Fig. 1:**
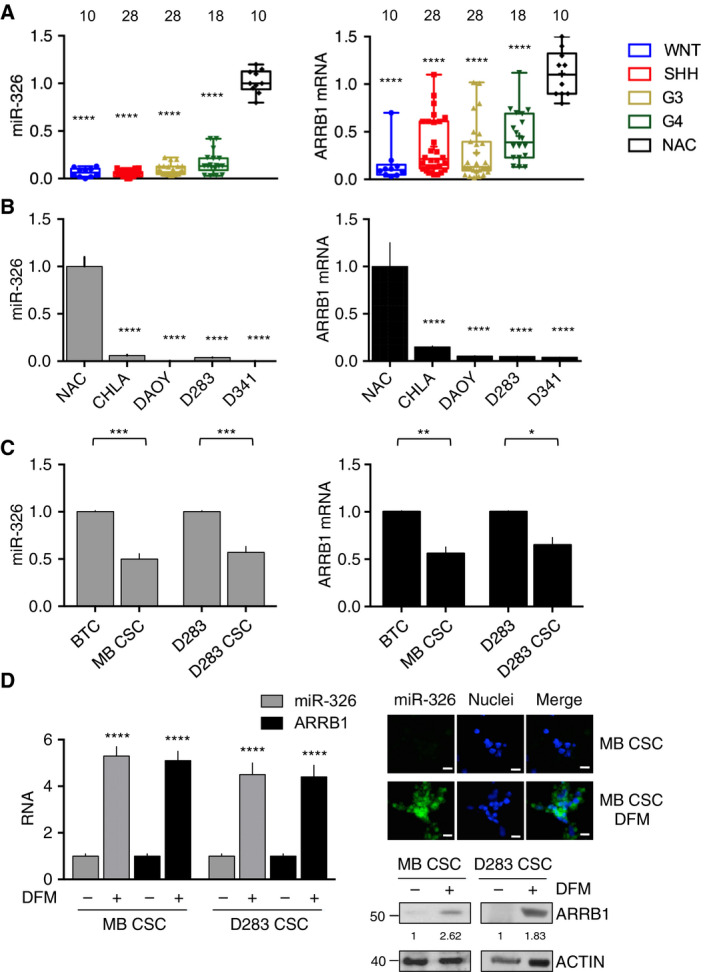
*miR‐326* and *ARRB1* under‐expression in MBs and MB cells. (A) qRT‐PCR showed markedly reduced *miR‐326* and *ARRB1* expression in the 84 MBs of cohort 1 vs. normal adult cerebellum (NAC, control) (*miR‐326*: WNT/SHH/G3/G4 vs. NAC *P* < 0.0001; *ARRB1*: WNT/SHH/G3/G4 vs. NAC *P* < 0.0001). Numbers of samples tested are indicated above columns. (B) qRT‐PCR revealed *miR‐326* and *ARRB1* mRNA levels in four MB cell lines. These levels were significantly lower than those in NAC (control) (miR‐326: CHLA, DAOY, D283, D341 vs. NAC *P* < 0.0001; ARRB1: CHLA, DAOY, D283, D341 vs. NAC *P* < 0.0001). (C) *miR‐326 and ARRB1* transcript levels in CSCs derived from primary MBs (cohort 1, MB CSC_1–6_) and D283 cells (D283 CSCs) (see Fig. S2) and those found in their respective bulk tumor cell (BTC) populations (*miR‐326*: MB CSC_1–6_ vs. BTC_1–6_ *P* = 0.0002; D283 CSC vs. D283 *P* = 0.0005; *ARRB1*: MB CSC_1–6_ vs. BTC_1–6_ *P* = 0.0025; D283 CSC vs. D283 *P* = 0.0105). (D) Mean miR‐326 and ARRB1 expression in MB CSCs and D283 CSCs before and after differentiation triggered by transfer from stem cell to differentiation medium (DFM). Left panel: *miR‐326* and *ARRB1* expression assessed by qRT‐PCR (*n* = 7) (MB CSC: *miR‐326* and *ARRB1*: DFM+ vs. DFM− *P* < 0.0001; D283 CSC: *miR‐326* and *ARRB1*: DFM+ vs. DFM− *P* < 0.0001). Center panel: fluorescence *in situ* hybridization assessment of miR‐326 (representative images, scale bar, 5 µm); Right panel: ARRB1 expression assessed by western blotting. Error bars represent standard deviation from the means. Statistics: One‐way ANOVA and two‐way ANOVA, *P < 0.05; **P < 0.01; ***P < 0.001; ****P < 0.0001.

Expression of *miR‐326* and *ARRB1* was analyzed in two independent patient cohorts (*n* = 84, *n* = 437) of primary MBs and in MB cell lines. Significant under‐expression of both genes, relative to normal adult cerebellum (NAC), was found in tumor samples from both cohorts, as well as in MB cell lines (Fig. 1A,B, Table S1 and Fig. S1). CSCs from MBs and from D283, both characterized by high levels of stemness markers, CD133 and NANOG (Fig. S2), were also investigated for the expression of miR‐326 and ARRB1. As shown in Fig. 1C, these cells expressed lower levels of both genes than their respective bulk tumor cell populations (BTC), considered as the starting population for primary MBs and the ATCC cell culture method for D283, respectively. Transfer of CSCs to differentiation medium (DFM) was accompanied by significantly upregulated expression of miR‐326 and ARRB1 at both the transcriptional and protein levels (Fig. 1D). These findings indicate that miR‐326 and ARRB1 show the same expression pattern and that their markedly downregulated expression is a feature of MB cells, including their CSC subset. Therefore, this hallmark might conceivably play important role(s) in MB onset and maintenance.

### 3.2 Epigenetic regulation of *miR‐326* and *ARRB1* expression in MB

**Fig. 2:**
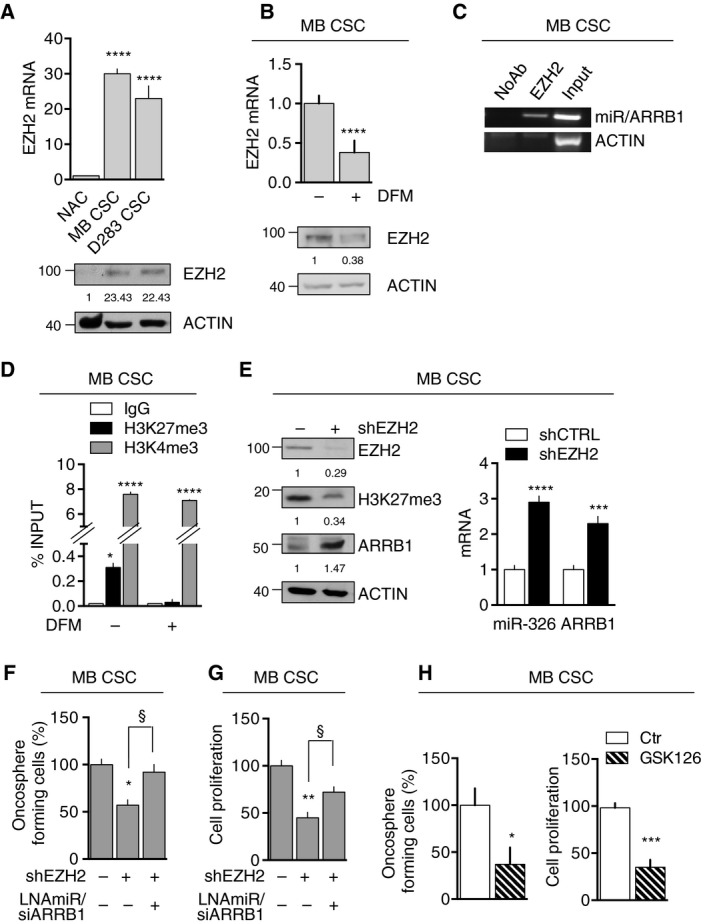
EZH2‐dependent regulation of miR‐326 and ARRB1. (A) EZH2 expression mRNA (top) and protein (bottom) in NAC (control), MB CSC and D283 CSC (vs. NAC: MB CSC *P* < 0.0001; D283 CSC *P* < 0.0001). (B) EZH2 expression (mRNA and protein) in MB CSCs (MB CSC_1–3_) before and after transfer to DFM. ACTIN: loading control (DFM+ vs. DFM−: *P* < 0.0001). (C) ChIP of MB CSC (MB CSC_1–3_) lysates precipitated with anti‐EZH2 antibody or (control) no antibody (NoAb). Eluted DNA was amplified by PCR using primers specific for the miR‐326/ARRB1 regulatory region. ACTIN (shown) and ARRB1 exon 5 and 11 primers (not shown) were used as endogenous nonenriched control regions. (See Table S2 for primer details.) (D) ChIP performed using anti‐H3K27me3 and anti‐H3K4me3 antibodies showing presence of repressive and activating histone marks on the *miR‐326* and *ARRB1* regulatory region (MB CSC_1–4_) (in SCM vs. No: H3K27me3 *P* = 0.017; H3K4me3 *P* < 0.0001, in DFM vs. No: HeK4me3: *P* < 0.0001). (E) MB CSCs were transduced with lentiviruses harboring EZH2‐specific shRNA (shEZH2) or (controls) shScramble (shCTRL). *Left*: Immunoblots showing EZH2, total H3K27me3, and ARRB1 protein levels. *Right*: *ARRB1* mRNA and *miR‐326* levels measured by qRT‐PCR (MB CSC_1–4_) (*miR‐326*: shEZH2 vs. shCTRL *P* < 0.0001; *ARRB1*: shEZH2 vs. shCTRL *P* = 0.0007). (F, G) MB CSCs were transduced with shEZH2, with or without ARRB1‐specific siRNA (siARRB1) plus LNAmiR‐326, and assayed for (F) self‐renewal, reflected by oncosphere formation (MB CSC_1–3_) (vs. shEZH2−LNAmiR/siARRB1−: shEZH2+ *P* = 0.011; shEZH2+LNAmiR/siARRB1+ *P* = 0.029) (G) and proliferation [MTT assay (MB CSC_1–4_), (vs. shEZH2−LNAmiR/siARRB1−: shEZH2+ *P* = 0.0016; shEZH2+LNAmiR/siARRB1+ *P* = 0.048)]. (H) Oncosphere formation (GSK126 vs. Ctr *P* = 0.013) (left) and proliferation MTT assay (GSK126 vs. Ctr *P* = 0.003) (right) of MB CSCs (MB CSC_1–4_) treated with GSK126 (5 µm for 48 h). All data represent means ± SD from at least three independent experiments. Statistics: Wilcoxon signed‐rank test for paired data, one‐way ANOVA and two‐way ANOVA, *P < 0.05; **P < 0.01; ***P < 0.001; ****P < 0.0001; ^§^< 0.05 vs. indicated controls.

We hypothesized that histone methyltransferase enhancer of zeste homolog 2 (EZH2) might play a role in the regulation of miR‐326 and ARRB1 in MBs. Indeed, EZH2 was shown to be overexpressed in MBs [6, 43, 44, 45, 46] and its inhibition is known to significantly disrupt CSC maintenance in MB and other brain tumors [38, 44, 47]. As shown in Fig. 2A and Fig. S3, our primary MBs and MB CSCs exhibited EZH2 protein and mRNA levels that were indeed significantly higher than those found in NAC. Levels were particularly high in MB CSCs, and they dropped significantly when CSCs were transferred to DFM (Fig. 2B). Chromatin immunoprecipitation (ChIP) experiments revealed recruitment of EZH2 to the *miR‐326/ARRB1* regulatory region in MB CSCs (Fig. 2C). This first set of experiment allowed us to identify EZH2 as a direct regulator of *miR‐326* and *ARRB1*.

EZH2 catalyzes the tri‐methylation of histone H3 at lysine 27 (H3K27me3) [48], a transcription‐repressive chromatin mark. It is frequently found together with the transcription‐activating mark tri‐methylation of histone H3 at lysine 4 (H3K4me3) and together they are named ‘bivalent domain’ [49]. The bivalency of the promoter allows developmental genes to be rapidly activated by the removal of H3K27me3 [50, 51, 52, 53]. Bivalent domains have also been found in cancer, where they can suppress the expression of tumor suppressors [54, 55].

We therefore examined the *miR‐326/ARRB1* regulatory region in MB CSCs for signs of bivalency. Indeed, this region displayed clear evidence of both H3K27me3 and H3K4me3 (Fig. 2D), furthermore the differentiation stimulus was associated with a marked increase in the H3K4me3:H3K27me3 ratio and diminished EZH2 binding of the *miR‐326/ARRB1* regulatory region (Fig. S4).

To further investigate EZH2's role in miR‐326 and ARRB1 suppression, we inhibited EZH2 in MB CSC by short hairpin‐mediated silencing (shEZH2) or an EZH2 pharmacological inhibitor, GSK126, recently used in our laboratory in MB [56]. As shown in Fig. 2E, EZH2 knockdown led to a marked decrease in the levels of H3K27me3 in transduced cells. EZH2‐depleted MB CSCs also exhibited upregulated expression of both ARRB1 and miR‐326 and decreased capacities for self‐renewal and proliferation (Fig. 2E–G). When the knockdown of EZH2 was accompanied by the combination of ARRB1‐specific siRNAs and locked nucleic acid (LNA)‐based miR‐326, thus preventing their shEZH2‐mediated upregulation, the effects on oncosphere formation and proliferation were abolished (Fig. 2F,G), suggesting that miR‐326 and ARRB1 are downstream mediators of the anti‐tumorigenic effects of EZH2 depletion in MB CSCs. Finally, GSK126‐dependent reactivation of miR‐326 and ARRB1 expression impaired both MB CSCs' oncosphere formation and proliferation (Fig. 2H).

Collectively, these results show that miR‐326 and ARRB1 are controlled by a bivalent domain, since H3K27me3 repressive mark is found at their regulatory region together with the activation‐associated H3K4me3 mark.

### 3.3 *In vitro* biological effects of ectopic miR‐326 and ARRB1 expression

**Fig. 3:**
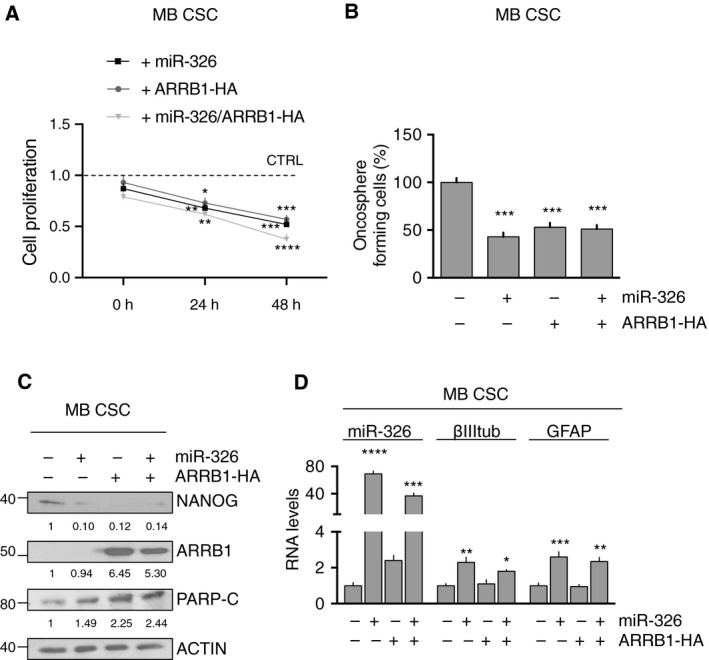
Biological effects of ectopic expression of miR‐326 and ARRB1 in MB CSCs. (A–C) MB CSCs were assayed 48 h after transfection with *miR‐326* and *ARRB1*, individually or combined. Mock‐transfected cells served as controls. Ectopic expression of *ARRB1* and/or *miR‐326* in these cells (A) reduced proliferation (MTT assay) (24 h: vs. CTRL: miR‐326+ *P* = 0.0072; ARRB1‐HA+ *P* = 0.0245; miR‐326+ARRB1‐HA+ *P* = 0.0015; 48 h: vs. CTRL: miR‐326+ *P* = 0.001; ARRB1‐HA+ *P* = 0.0005; miR‐326+ARRB1‐HA+ *P* < 0.0001), (B) diminished the frequency of oncosphere‐forming cells (vs. miR‐326‐ARRB1‐HA−: miR‐326+ *P* = 0.001; ARRB1‐HA+ *P* = 0.0005; miR‐326+ARRB1‐HA+ *P* = 0.0003), (C) decreased NANOG expression and increased the expression of PARP‐C (miR‐326 levels: miR‐326+ *P* < 0.0001; miR‐326+ARRB1‐HA+ *P* = 0.0002 vs. miR‐326‐ARRB1‐HA−). (D) qRT‐PCR revealed significantly increased expression of neuronal and glial differentiation markers (*βIII tubulin* and *GFAP*, respectively) only in MB CSCs overexpressing *miR‐326* (alone or with *ARRB1*) (vs. Mock βIII tubulin: miR‐326 *P* = 0.0027; miR‐326 and ARRB1 *P* = 0.046, GFAP: miR‐326 *P* = 0.0002; miR‐326 and ARRB1 *P* = 0.011). Data represent means ± SD from five independent experiments. Statistics: One‐way ANOVA and two‐way ANOVA, *P < 0.05; **P < 0.01; ***P < 0.001; ****P < 0.0001 vs. indicated controls.

We then explored the biological consequences of low levels of miR‐326 and ARRB1 through their ectopic re‐expression in MB CSCs. Overexpression of miR‐326 and ARRB1, both singularly and combined, caused significantly reduced proliferation (Fig. 3A), reduced stemness features, as reflected by suppressed oncosphere formation (Fig. 3B) and markedly downregulated expression of the stemness marker NANOG (Fig. 3C). In addition, we investigated apoptosis and differentiation. Our results show that ARRB1, both alone and in combination with miR‐326, was able to increase levels of cleaved poly (ADP‐ribose) polymerase [PARP‐C], a marker of apoptosis (Fig. 3C), while overexpression of miR‐326, alone and in combination with ARRB1, significantly upregulated the expression of neuronal (βIII tubulin) and glial (GFAP) differentiation markers (Fig. 3D). These findings highlight the tumor‐promoting effects of downregulated expression of miR‐326 and ARRB1 in MB cells, whose biological convergence includes impairment of apoptosis and differentiation, along with enhancement of the CSC component's pluripotency and clonogenicity.

### 3.4 E2F1 is overexpressed in MBs and post‐transcriptionally regulated by miR‐326 and ARRB1

**Fig. 4:**
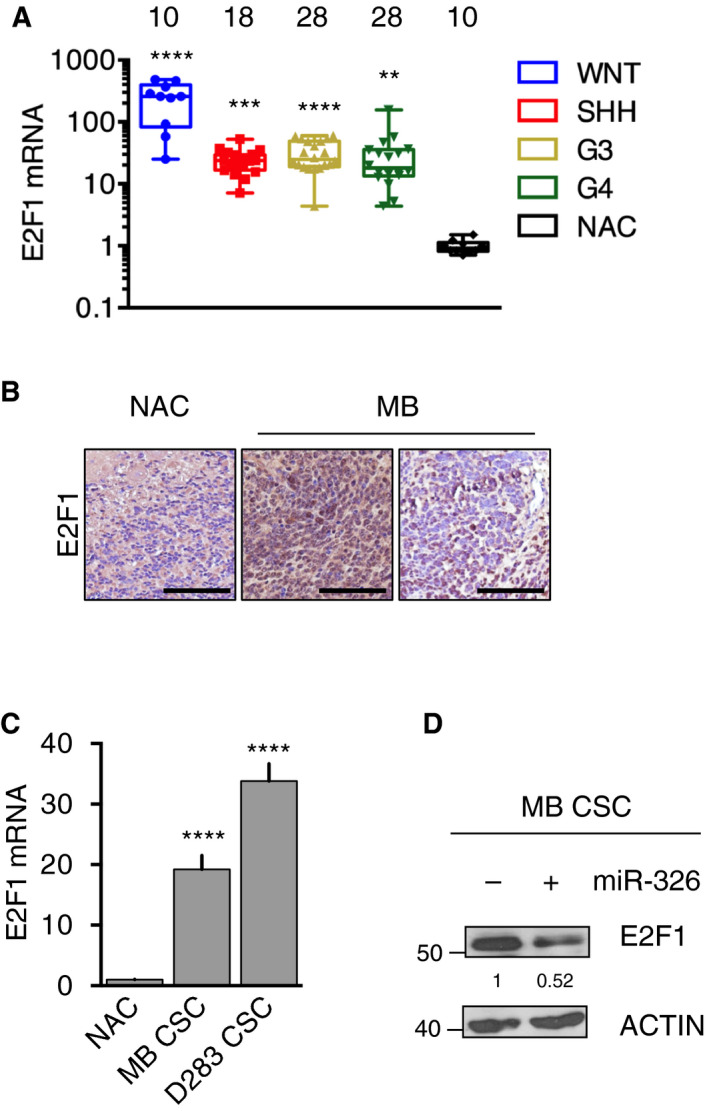
E2F1 is overexpressed in MBs and is a direct target of miR‐326. (A) qRT‐PCR analysis of the 84 primary MBs of cohort 1 disclosed significant tumor‐related increases (vs. NAC) in the transcription of the miR‐326 target *E2F1* (vs. NAC: WNT *P* < 0.0001; SHH *P* = 0.0007; G3 *P* < 0.0001; G4 *P* = 0.0024). Numbers on top indicate number of samples tested. (B) IHC staining for E2F1 in sections of NAC and representative cohort 1 MB samples. Magnification 40×, Scale: 100 µm. (C) *E2F1* mRNA levels in NAC (control) MB CSC and D283 CSC (vs. NAC: MB CSC *P* < 0.0001; D283 CSC *P* < 0.0001). (D) Representative immunoblot showing significantly decreased E2F1 expression in MB CSCs after ectopic *miR‐326* expression (vs. Mock, mock‐transfected controls, *P* < 0.0001). Data represent means ± SD from three independent experiments. Statistics: One‐way ANOVA test and Wilcoxon signed‐rank test for paired data, **P < 0.01; ***P < 0.001; ****P < 0.0001 vs. indicated controls.

Next, we explored a possible mechanism underlying the effects of miR‐326 and ARRB1 in MB. Previous evidences led us to focus our attention on E2F1, a validated target of miR‐326 [57], whose levels are reduced by EZH2 silencing in MB [44] and a more recent evidence reported convergence of prognostic signaling pathways on E2F1 in MB [58]. Furthermore, it is interesting to note that an E2F1 transgenic mouse model was shown to develop brain tumors, including MBs [59]. As shown in Fig. 4A,B and Fig. S5, E2F1 proved to be highly overexpressed in MBs of cohorts 1 and 2. It was also expressed at high levels in MB CSC (Fig. 4C) and levels clearly declined after re‐expression of *miR‐326* (Fig. 4D).

Thus, the upregulation of E2F1 in MBs appears to depend at least in part on the low levels of miR‐326. Since *miR‐326* expression is co‐regulated with that of *ARRB1*, we wondered whether the under‐expression of ARRB1 in MBs could also be linked to high expression levels of E2F1. E2F1 plays roles in cell growth as well as in apoptosis [60]; its selectivity for pro‐apoptotic target genes (e.g., *TP73, CASP3, CASP7*) is determined mainly by its post‐translational acetylation, which can be catalyzed by the acetyl‐transferase p300 [40, 61, 62]. Interestingly, p300 is known to form complexes with ARRB1 [25, 31, 63, 64]. Thus, we reasoned that this type of interaction might be involved in the anti‐proliferative and pro‐apoptotic effects observed when ARRB1 was ectopically expressed in MB CSCs, as shown in Fig. 3C.

**Fig. 5:**
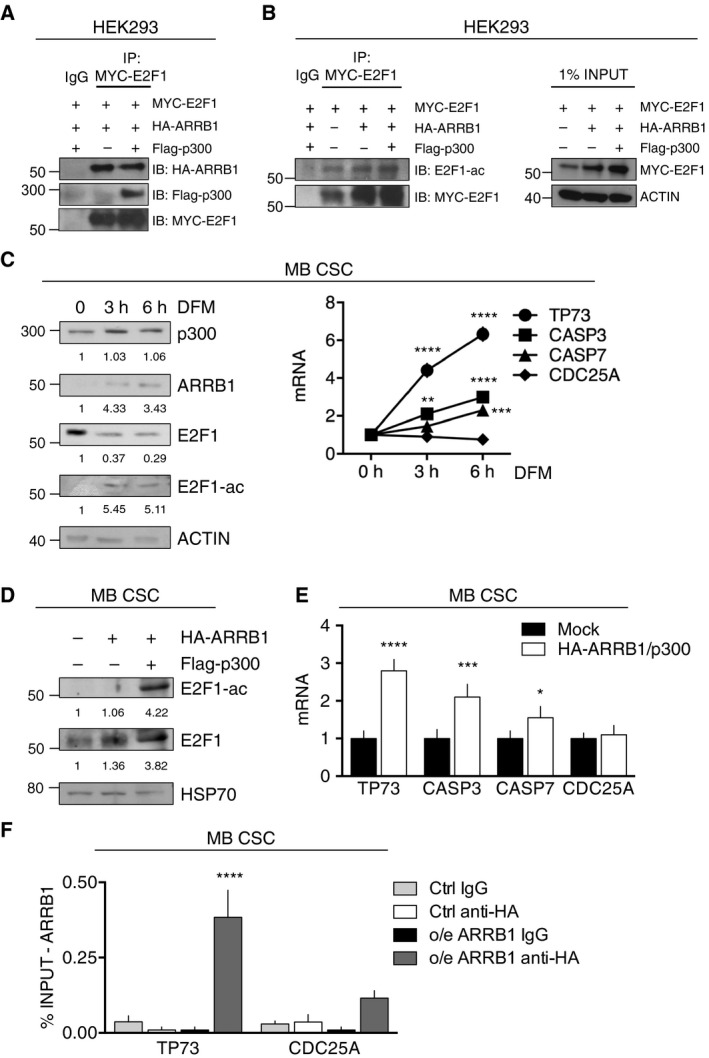
ARRB1 modulates E2F1 acetylation and functions. (A, B) HEK293 cells were transfected with plasmids expressing MYC‐*E2F1*, HA‐*ARRB1* and/or Flag‐*p300*, as indicated. Total cell extracts were incubated with anti‐MYC antibody (IP), or IgG (negative control) and immunoprecipitated with protein A‐coupled beads. (A) Immunoblotting (IB) of the MYC‐E2F1 precipitate revealed the co‐presence of HA‐ARRB1 and Flag‐p300. (B) Left: Total extracts of HEK293 cells transfected with the indicated plasmids were immunoprecipitated (IP) with anti‐MYC or IgG and immunoblotted (IB) with anti‐acetylated‐E2F1 (E2F1‐ac) and anti‐MYC. Right panel: 1% of the immunoprecipitated cell lysates (INPUT) was immunoblotted with anti‐MYC and anti‐actin (loading control). (C) Untransfected MB CSCs (MB CSC_1–3_) were assayed under basal conditions (growth in SCM) (0 h, control) and after 3 or 6 h of growth in DFM: (left) immunoblot analysis of endogenous expression ARRB1, p300, E2F1, and E2F1‐ac levels and (right) qRT‐PCR assessment of E2F1 pro‐apoptotic (*TP73*) and pro‐proliferative (*CDC25A*) target gene transcription (vs. 0 h: TP73 3 h *P* < 0.0001, 6 h *P* < 0.0001; CASP3 3 h *P* = 0.0023, 6 h *P* < 0.0001; CASP7 6 h *P* = 0.0004). (D) MB CSCs transfected with HA‐*ARRB1* or HA‐*ARRB1* plus Flag‐*p300* were assayed for endogenous expression of E2F1 and E2F1‐ac (immunoblotting). (E) MB CSCs (MB CSC_1–4_) transfected with HA‐*ARRB1* plus Flag‐*p300* were assayed for expression of pro‐apoptotic and pro‐proliferative E2F1 target genes (qRT‐PCR) (vs. Mock: TP73 *P* < 0.0001; CASP3 *P* = 0.0002; CASP7 *P* = 0.026). (F) Anti‐HA‐ARRB1 ChIP in MB CSCs (MB CSC_1–4_) 24 h after HA*‐ARRB1* overexpression (o/e). Eluted DNA was qPCR‐amplified using specific primers for the regulatory regions of *TP73 and CDC25A* (o/e ARRB1‐HA: TP73 *P* < 0.0001). Data represent means ± SD from at least 3 independent experiments. Statistics: One‐way ANOVA and two‐way ANOVA, *P < 0.05; **P < 0.01; ***P < 0.001; ****P < 0.0001 vs. indicated controls.

To explore this hypothesis, we first performed immune‐precipitation (IP) experiments on HEK293 cells transfected with plasmids expressing HA‐tagged *ARRB1*, Flag‐tagged *p300* and MYC‐tagged *E2F1*. As shown in Fig. 5A, ARRB1 clearly formed a complex with E2F1 and p300 and this interaction was associated with an appreciable increase in E2F1 acetylation (Fig. 5B). IP experiments were also performed on endogenous proteins in GCPs [14, 65], where both ARRB1 and E2F1 are expressed and ARRB1 has been shown to contribute to the coordinated sequence of signaling regulating the proliferation, differentiation, and death of GCPs [25, 26]. Figure S6 shows the co‐immunoprecipitation of ARRB1 and E2F1, accompanied by the acetylation of E2F1. This phenomenon was further strengthened by the stimulus of SHH, that physiologically drives GCPs proliferation, while ARRB1 mediates an anti‐proliferative and pro‐apoptotic effect in the presence of SHH [25].

Next, we analyzed *endogenous* E2F1 levels in MB CSCs before and after transfer to DFM, which restores the expression of miR‐326 and ARRB1 as shown in Fig. 1D. As shown in Fig. 5C, CSCs expressed high levels of E2F1, which was all unacetylated in spite of the presence of p300. However, when ARRB1 expression was induced by the differentiation stimulus, appreciable levels of acetylated E2F1 appeared in the cells, along with increased levels of p300. These changes markedly upregulated the transcription of E2F1 target genes with pro‐apoptotic functions (mainly *TP73*), whereas transcript levels for the cell‐cycle progression marker *CDC25A* remained stable (Fig. 5C). The mechanistic importance of ARRB1 expression in these pro‐apoptotic effects was confirmed when Flag‐*p300* and HA‐*ARRB1* were overexpressed in CSCs (Fig. 5D,E). In chromatin immune‐precipitation (ChIP) studies, the ARRB1 ectopically expressed in these cells interacted preferentially with the promoter region of *TP73* (as compared with that of *CDC25A*) (Fig. 5F). Taken together, these data demonstrate that under‐expression of miR‐326 and ARRB1 in MB CSCs exert distinct effects on E2F1, which finally promote proliferation and survival. Specifically, the loss of miR‐326 de‐represses the expression of E2F1, and the loss of ARRB1 prevents its acetylation, which is necessary for the transcription factor's pro‐apoptotic effects.

### 3.5 *In vivo* biological effects of EZH2 knockdown and pharmacological inhibition in MB CSCs

To confirm the above‐reported effects of EZH2 in an *in vivo* setting, we compared growth rates of xenograft tumors (XTs) transduced with shRNA directed against *EZH2* or with sh*Scramble* (XT‐shEZH2 and XT‐Mock, respectively). We generated orthotopic XTs in immunocompromised mice, as shown in Fig. S7, XTs generated with D283 CSCs were morphologically comparable to those generated with primary MB CSC_1_ or MB CSC_3_. However, since D283 CSC‐generated tumors formed more rapidly (Fig. S7), they were used in all subsequent experiments.

**Fig. 6:**
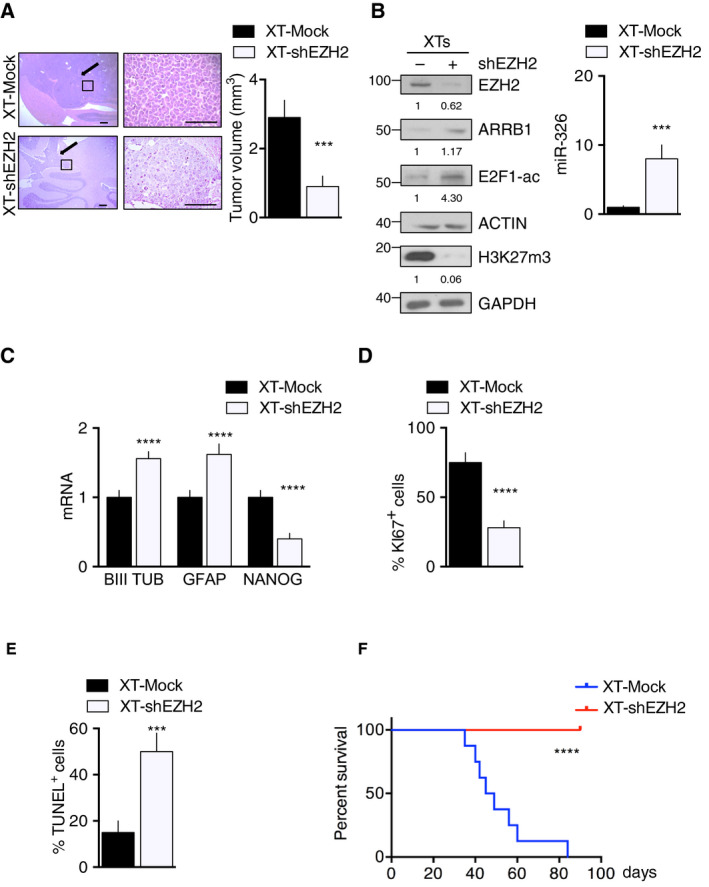
*In vivo* reactivation of miR‐326 and ARRB1 expression by EZH2 knockdown in MB CSCs. Xenograft tumors (XTs) generated in immunocompromised mice using D283 CSC transduced with lentiviral shEZH2 (XT‐shEZH2) or sh*Scramble* (XT‐Mock). (A) Left: Representative images of H&E‐stained, largest‐diameter XT sections. Arrows indicate tumor masses. Magnification: 40× (left column), 400× (right). Scale bar, 100 µm. Right: Bar graphs showing XT volumes on postimplantation day 28. Bars represent means (SD), *P* = 0.0005. (B, C) XT‐shEZH2 and XT‐Mock expression of (B) (left) EZH2, ARRB1, E2F1‐ac and H3K27me3 proteins (ACTIN, GAPDH: loading control), (left) miR‐326 *P* = 0.0004, (C) mRNA for markers of differentiation (neuronal: βIIItub *P* < 0.0001; astrocytic: GFAP *P* < 0.0001) and stemness (NANOG *P* < 0.0001). (D, E) XT‐shEZH2 and XT‐Mock were assayed for (D) cell proliferation (Ki67 IHC) *P* < 0.0001 and (E) apoptosis (TUNEL assay positive cells) *P* = 0.0003. Data represent means ± SD from three independent experiments. Statistics: Wilcoxon signed‐rank test for paired data and two‐way ANOVA test, ***P < 0.001, ****P < 0.0001 vs. relative controls. (F) Kaplan–Meier analysis of survival for mice bearing XT‐shEZH2 vs. XT‐Mock (*n* = 8 per group), *P* < 0.0001.

As shown in Fig. 6A and Fig. S10A, mean XT‐Mock volumes significantly exceeded those of XTs generated with EZH2‐depleted D283 CSCs (XT‐shEZH2). The genetic suppression of EZH2 activity in the XT‐shEZH2 cells was accompanied by substantial downregulation of H3K27me3, upregulation of ARRB1 and miR‐326 expression and induction of E2F1 acetylation (E2F1‐ac) (Fig. 6B). XT‐shEZH2 also exhibited upregulated expression of differentiation markers and attenuated stemness features (Fig. 6C), decreased proliferation (Fig. 6D) and significantly increased apoptosis (Fig. 6E). These tumor‐inhibiting effects were also reflected by significantly improved survival of XT‐shEZH2‐bearing mice (Fig. 6F).

We proceeded to evaluate the pharmacological inhibition of EZH2 in xenograft tumors (XTs) generated in D283 CSC‐XTs using EZH2 inhibitor, MC3629, described in [38]. Cells were implanted, and after 7 days, mice were separated into two groups: mice that received MC3629 (XT‐MC3629) or vehicle (XT‐Mock) for 21 days. Pharmacological inhibition of EZH2 in XTs resulted in increase of miR‐326 levels and was accompanied by upregulation of ARRB1 and acetylated E2F1 (E2F1‐ac) and decrease of EZH2 levels (Fig. S8).

Collectively, these results confirm that EZH2 is responsible for the impaired expression of miR‐326 and ARRB1 in MBs, which eliminates important checks of tumor growth.

### 3.6 *In vivo* biological effects of ectopically expressed miR‐326 and ARRB1

**Fig. 7:**
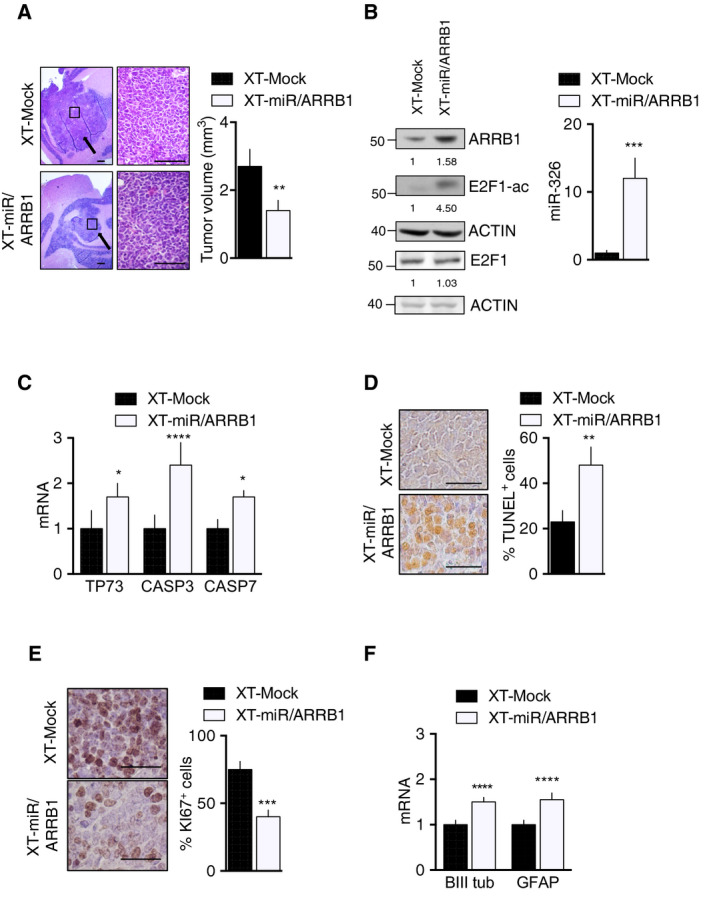
Ectopic expression of miR‐326 and ARRB1 inhibits MB growth *in vivo*. Orthotopic XTs were generated in immunocompromised mice by injection of D283 CSCs transduced with separate vectors overexpressing *miR‐326* and *ARRB1* (XT‐miR/ARRB1) or empty vector (XT‐Mock, controls). (A) Representative images of H&E‐stained XTs (largest‐diameter sections). Arrows indicate tumor masses. Bar graph: Mean XT volumes at animal sacrifice (28 days postimplantation) *P* = 0.0043. XTs were assayed for (B) ARRB1 and E2F1‐ac protein levels (left), *miR‐326* levels (right) *P* = 0.0001; (C) expression of pro‐apoptotic E2F1 target genes (qRT‐PCR) (TP73 *P* = 0.0226; CASP3 *P* < 0.0001; CASP7 *P* = 0.002); (D) apoptosis (TUNEL assay, *P* = 0.0018); (E) cell proliferation (Ki67 IHC, *P* = 0.0001); (F) and expression of neuronal and glial differentiation markers (βIIItub *P* < 0.0001 and GFAP *P* < 0.0001, respectively). Magnification in panels A, D, and E: 4×, 40×, 63×; all scale bars, 100 µm. Data represent means ± SD from eight independent experiments. Statistics: Wilcoxon signed‐rank test for paired data and two‐way ANOVA, *P < 0.05; **P < 0.01; ***P < 0.001; ****P < 0.0001 vs. indicated controls.

We assessed the functional *in vivo* relevance of miR‐326 and ARRB1 re‐expression. D283 CSC‐XTs overexpressing miR‐326 and ARRB1 (XT‐miR‐326 and ARRB1) were significantly smaller than those generated with mock‐transfected cells (XT‐Mock), Fig. 7A and Fig. S10B. In addition, the significantly higher levels of ARRB1 and miR‐326 were accompanied by the appearance of acetylated E2F1 (E2F1‐ac) (Fig. 7B), decreased *EZH2* levels (Fig. S9), upregulated transcription of pro‐apoptotic E2F1 target genes with marked increase in apoptosis (Fig. 7C,D), diminished proliferation (Fig. 7E), and increased differentiation (Fig. 7F). These data confirm that MB‐associated under‐expression of miR‐326 and ARRB1 exerts tumor‐promoting effects *in vivo* as well as *in vitro*.

## 4 Discussion

A better understanding of the molecular mechanisms characterizing MB progression is essential for developing safer and more effective therapies for patients with these tumors [66]. Of note, recently molecular mechanisms involving microRNA contributing to brain tumors progression, specifically glioblastoma, have been reported, with important therapeutic implication [67].

Some reports suggest E2F1's involvement in MB. First, a transgenic mouse model expressing E2F1 in GFAP‐expressing cells (thus including neural precursors) developed brain tumors, including MBs [59].

In addition, E2F1 in MB regulates lipogenic enzymes, controlling cell proliferation and tumor aggressiveness [68], and its overexpression is also a documented feature of self‐renewing neural stem cells, where it declines markedly when these cells undergo differentiation [69].

Interestingly, we found that E2F1 is a validated target of miR‐326 [57] and indeed in our MB models loss of miR‐326 promotes cell‐cycle progression by upregulating its protein expression level.

The more complex tumor‐promoting effects of ARRB1 loss are related to the protein's importance for inducing E2F1 acetylation, a modification that ultimately leads to the transcriptional activation of E2F1's pro‐apoptotic target genes. ARRB1 is a key signal‐transducing element in several intracellular signaling pathways involved in cell development [70]. In neurons, ARRB1 translocates to the nucleus, where it associates with the transcription factor CREB and p300 acetyltransferase on the promoters of its target genes, directly enhancing their transcription [25, 63]. ARRB1, as miR‐326, has a role in neuronal differentiation; its upregulation in cerebellar GCPs and in neural stem cells halts proliferation and induces growth arrest [25, 26].

Moreover, ARRB1 expression inversely correlates with proliferation of GCPs derived from a transgenic mouse model involving RE1‐silencing transcription factor (REST), a transcriptional repressor of neuronal differentiation [32].

ARRB1 can directly modulate the expression of genes involved in diverse cell functions, including cell‐cycle arrest/differentiation, proliferation/survival, and apoptosis [71, 72]. As a result, the functional consequences of its interaction with gene promoters are cell‐type‐dependent.

For this reason, the fact that miR‐326 and ARRB1 appear to exert convergent tumor‐suppressive effects in human MBs is by no means incompatible with its demonstrated oncogenic effects in other cancer cells [71, 73, 74, 75, 76].

In keeping with the documented organ and site dependency of its over‐ and under‐expression, ARRB1 appears to play a tumor‐suppressor role in brain tumors.

Indeed, ARRB1 under‐expression has been documented (in some cases along with that of miR‐326) in adult and pediatric gliomas [23, 27, 28, 29, 30] and glioblastomas [23]. In line with this, we recently reported the ability of ARRB1 to regulate Hedgehog/Gli signaling via acetylation of Gli1 in the MB [31].

Looking for a regulatory mechanism of miR‐326 and ARRB1, we found that EZH2 is overexpressed in MBs [6, 43, 44, 45, 46] and in MB CSC [38, 44, 47].

Overexpression or activating mutations of EZH2 have been described in several malignancies [77], where they are usually associated with tumor aggressiveness, resistance to drug therapy and poor outcomes [78, 79, 80]. In adult and pediatric brain tumors, EZH2 expression also increases with tumor grade [81] and it is known to sustain self‐renewal in the cancer stem‐like cell population of glioblastoma [47, 82].

Genome‐wide exome sequencing studies revealed recurrent alterations involving EZH2 and several other genes that influence histone methylation, including those encoding the H3K4 methyltransferases, MLL2 and MLL3, and the H3K27me3 demethylases KDM6A and KDM6B in MBs [45, 46, 49, 83, 84, 85, 86]. EZH2 overexpression is associated with genomic gains of chromosome 7 in MBs, particularly (but not exclusively) in G3 and G4 tumors [38, 44, 45].

In the MB models, we analyzed the upregulated expression of EZH2 repressed that of miR‐326 and ARRB1, thereby favoring the maintenance of an undifferentiated CSC pool. Notably, significantly increased levels of H3K27me3 have already been reported in MBs [1, 46, 83].

Taken together, our data support previous reports on the potential value of EZH2 inhibition in the treatment of MB [38, 44], and they merit consideration in attempts to develop molecularly targeted therapies for more effective management of these tumors.

We show that both genetic and pharmacological abrogation of EZH2 expression restores miR‐326 and ARRB1 expression in MBs, including their CSCs component, blocks MB growth by limiting E2F1 pro‐proliferative activity, substantially decreasing the growth of MB cells both *in vitro* and *in vivo* and prolonging the survival of mice bearing tumors generated with MB CSC.

## 5 Conclusions

In this study, we identified a previously undescribed mechanism that promotes MB growth and maintains the CSC subpopulation in an undifferentiated state characterized by proliferation, enhanced self‐renewal and resistance to apoptosis.

With this work, we showed that under‐expression of miR‐326 and its host gene ARRB1 is a feature of primary human MBs, as well as of MB CSC cellular component.

We also described that EZH2 is responsible for the low expression of miR‐326 and ARRB1 in MBs, and this evidence has important implications for therapeutic strategies. Indeed, EZH2 inhibition restored miR‐326 and ARRB1 expression that in turn limited E2F1 pro‐proliferative activity with a final inhibition of MB growth in xenograft tumors.

Finally, our study highlights a novel E2F1‐critical role in MB progression with its pro‐proliferative activity maintained by the low levels of miR‐326 and ARRB1.

**Fig. 8:**
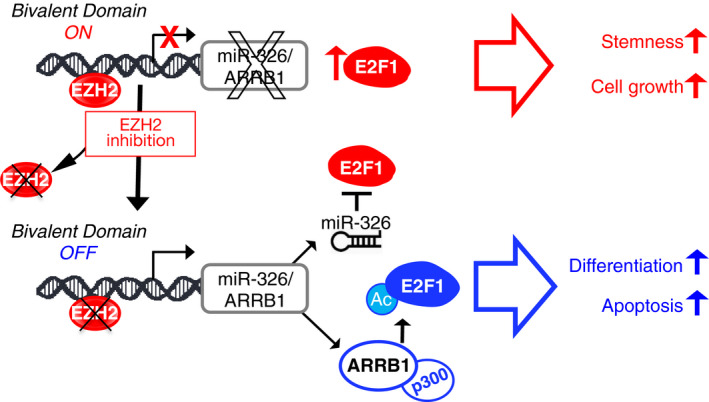
Impact of EZH2—miR‐326/ARRB1—E2F1 axis in MB CSCs. In MB CSCs, the *miR‐326* and *ARRB1* transcription unit remains in a poised state: ready to be transcribed thanks to the presence of the permissive (H3K4me3) chromatin mark, but prevented from doing so by the persistence/predominance of the repressive (H3K27me3) chromatin mark, which is catalyzed by the histone methyltransferase EZH2. In this state, the cells express high levels of nonacetylated E2F1 transcription factor, which favors their self‐renewal and proliferation. Reversal of the H3K4me3: H3K27me3 ratio de‐represses *miR‐326* and *ARRB1* transcription. Restoration of miR‐326 expression reduces the E2F1 levels. Re‐expression of ARRB1, in complex with p300, acetylates E2F1 (E2F1‐Ac), thereby redirecting the transcription factor's activity toward pro‐apoptotic gene targets (e.g., *TP73, CASP3, CASP7*).

Figure 8 outlines our findings in a model that shows how the loss of miR‐326 and ARRB1 cooperates on the expression and function of the E2F1 transcription factor, a key mediator of cell proliferation [60]. In the model, we also report that miR‐326 and ARRB1 are controlled by a bivalent domain, since the H3K27me3 repressive mark is found at their regulatory region together with the activation‐associated H3K4me3 mark and the domain is under the EZH2 control.

In conclusion, our findings highlight a novel molecular regulatory mechanism with potential therapeutic implication in MB.

## Conflict of interest

The authors declare no conflict of interest.

## Author contributions

The overall study was conceived and designed by EM, EF with important contributions from MK. EM, AP, NP, LA, GCatanzaro, and CS performed the majority of the *in vitro* experiments. EM and AP planned and performed *in vivo* studies and experimental treatments. EM, AP, ZMB, NP, and LA analyzed the in vitro data. SPF, MK, and FG performed and analyzed genes expressions in the clinical cohorts. AMastronuzzi, AC, SMP, MK, and FL provided human medulloblastoma cohorts and tissue. EM designed primers and performed qPCR experiments. EDS, GC, LDM, AV, and AMai generated various plasmid constructs and retroviruses used in this study. EM, AP, ZMB, and ML analyzed the data and performed statistical analyses. EM and EF wrote the manuscript with significant contributions from EDS and ML. All authors have read and approved the final submitted manuscript.

## Supporting information


**Fig. S1.** *miR‐326* and *ARRB1* expression in cohort 2 tumors.

**Fig. S2.** MB CSCs properties.

**Fig. S3.** EZH2 mRNA levels in MB samples and normal adult cerebella (NAC).

**Fig. S4.** Bivalency signs in MB CSCs miR‐326/ARRB1 regulatory region.

**Fig. S5.** E2F1 expression levels in cohort 2 of MB samples and normal adult cerebella (NAC).

**Fig. S6.** ARRB1 modulates E2F1 acetylation in granule cell precursors (GCPs).

**Fig. S7.** Characteristics of the orthotopic brain XTs generated in immunocompromised mice using MB CSCs.

**Fig. S8.** *In vivo* pharmacological inhibition of EZH2 in MB CSCs.

**Fig. S9.** Ectopic miR‐326 and ARRB1 expression inhibits MB cell growth *in vivo*.

**Fig. S10.** Hematoxylin and eosin staining images of XT *in vivo* experiments.

**Table S1.** Characteristics of cohort 1 tumors.

**Table S2.** List of primers used in Chromatin immunoprecipitation experiments.

**Appendix S1.** Materials and methods.
Supplementary Material

 