# Supplementary figures and images for: Downregulation of miR‐326 and its host gene β‐arrestin1 induces pro‐survival activity of E2F1 and promotes medulloblastoma growth
Source: Mol Oncol. 2020 Dec 31;15(2):523–42. doi: 10.1002/1878-0261.12800 (PMC7858128; doi:10.1002/1878-0261.12800)

Supplementary Figure 1. *miR-326* and *ARRB1* expression in cohort 2 tumors

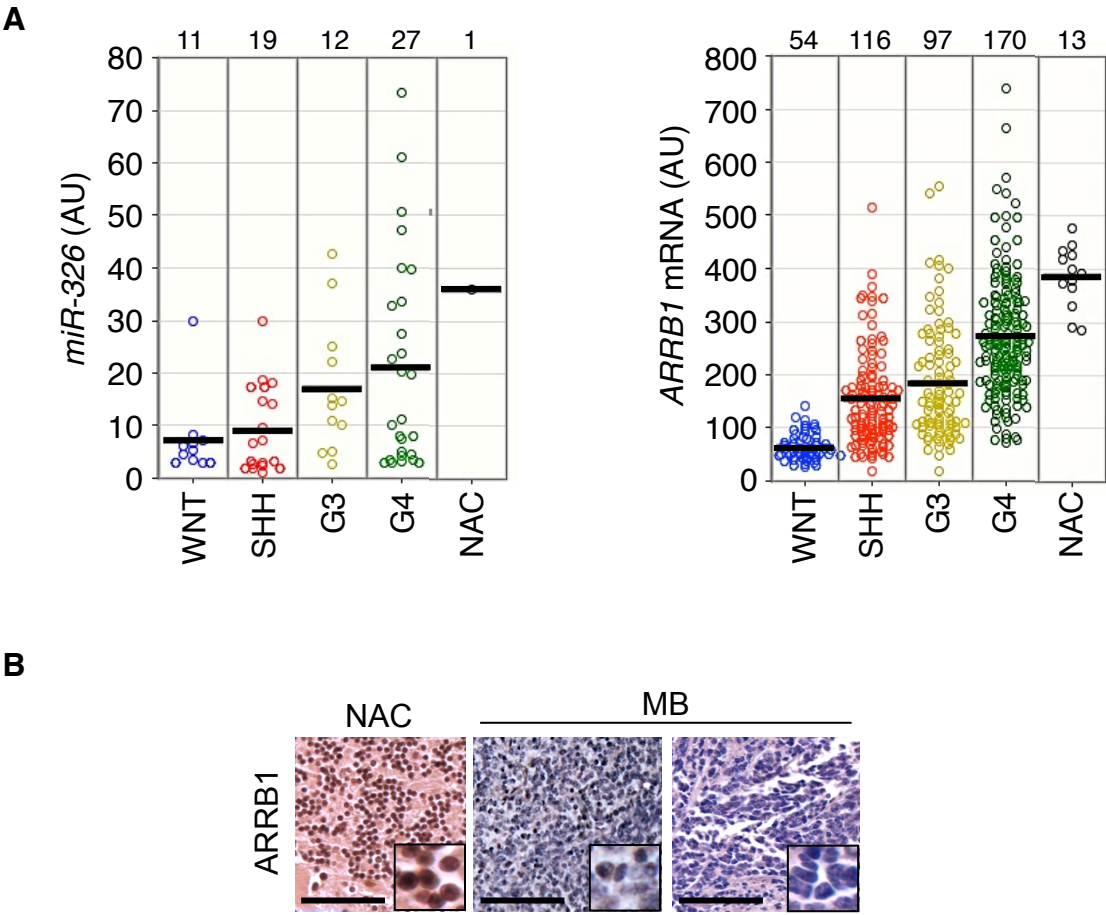

Supplement: Supplementary file 1 — Fig. S1. miR‐326 and ARRB1 expression in cohort 2 tumors. [file MOL2-15-523-s001.pdf]

Supplementary Figure 2. MB CSCs properties

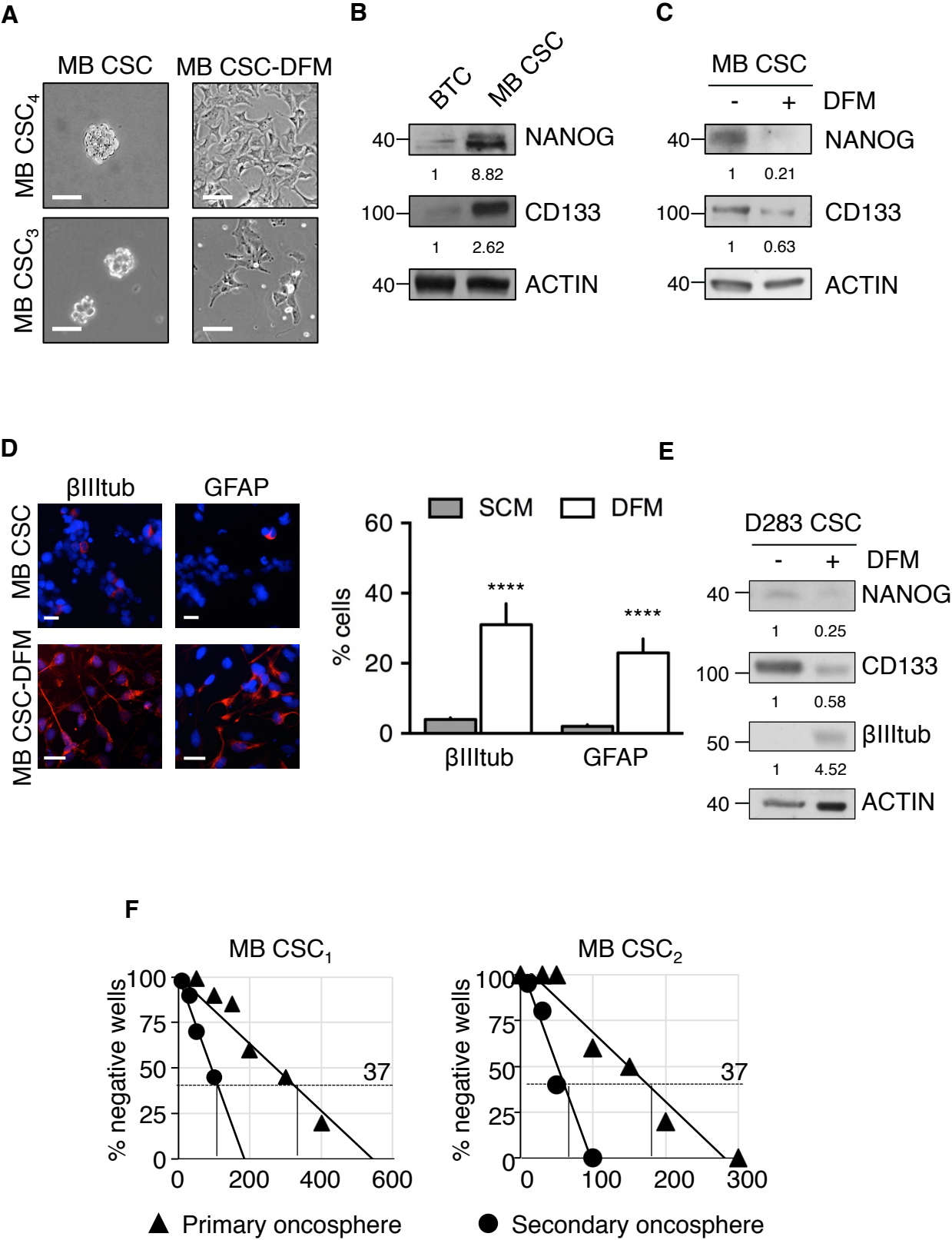

Supplement: Supplementary file 2 — Fig. S2. MB CSCs properties. [file MOL2-15-523-s002.pdf]

Supplementary Figure 3. EZH2 mRNA levels in MB samples and normal adult cerebella (NAC)

A

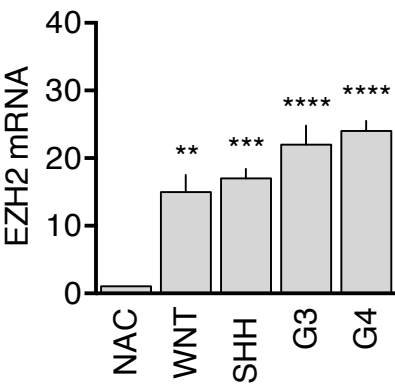

B

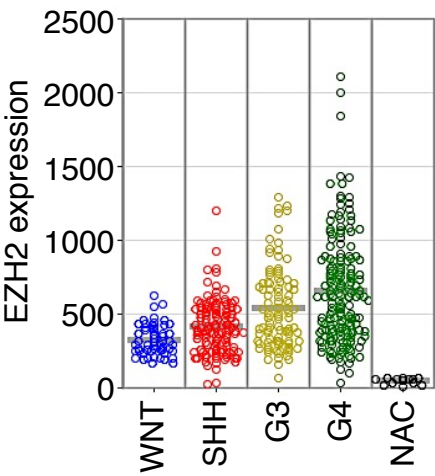

Supplement: Supplementary file 3 — Fig. S3. EZH2 mRNA levels in MB samples and normal adult cerebella (NAC). [file MOL2-15-523-s003.pdf]

Supplementary Figure 4. Bivalency signs in MB CSCs miR-326/ARRB1 regulatory region

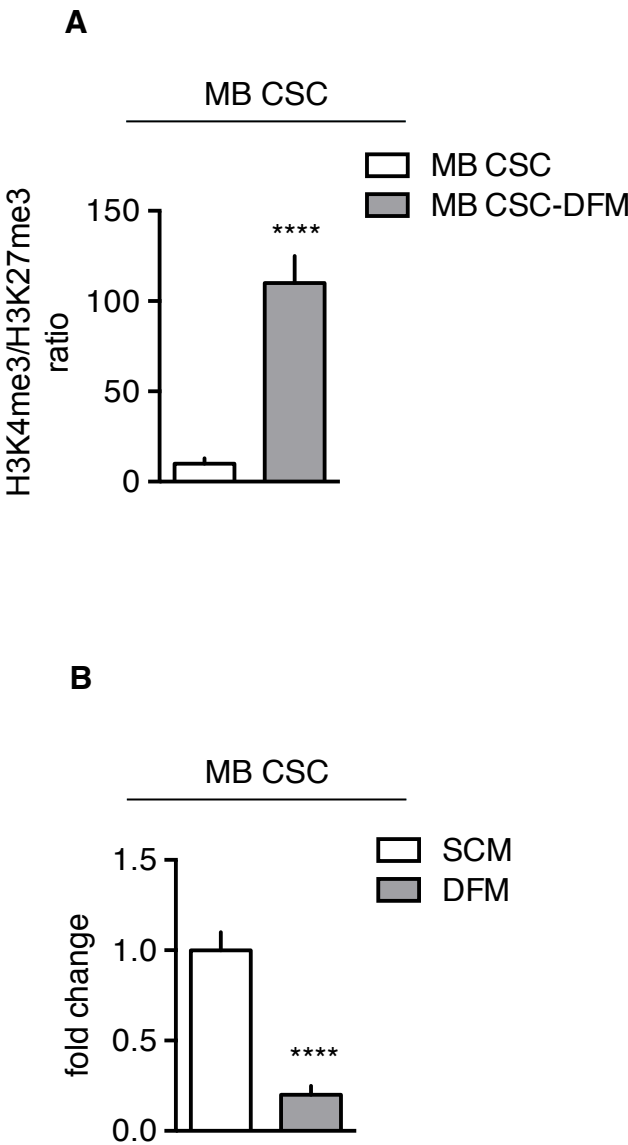

Supplement: Supplementary file 4 — Fig. S4. Bivalency signs in MB CSCs miR‐326/ARRB1 regulatory region. [file MOL2-15-523-s004.pdf]

Supplementary Figure 6. ARRB1 modulates E2F1 acetylation in granule cell precursors (GCPs)

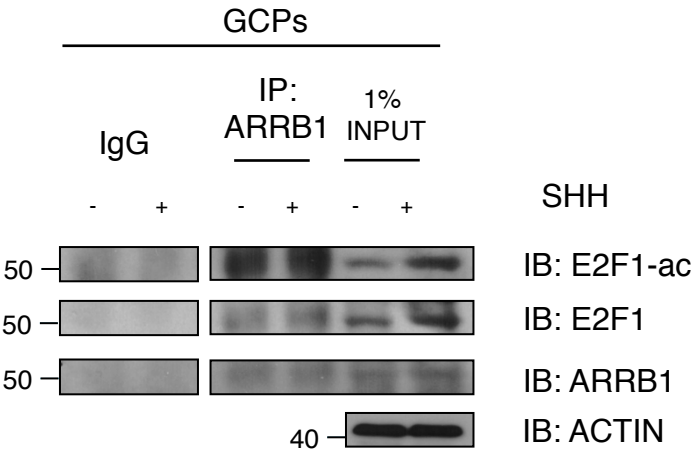

Supplement: Supplementary file 6 — Fig. S6. ARRB1 modulates E2F1 acetylation in granule cell precursors (GCPs). [file MOL2-15-523-s006.pdf]

Supplementary Figure 8. *In vivo* pharmacological inhibition of EZH2 in MB CSCs

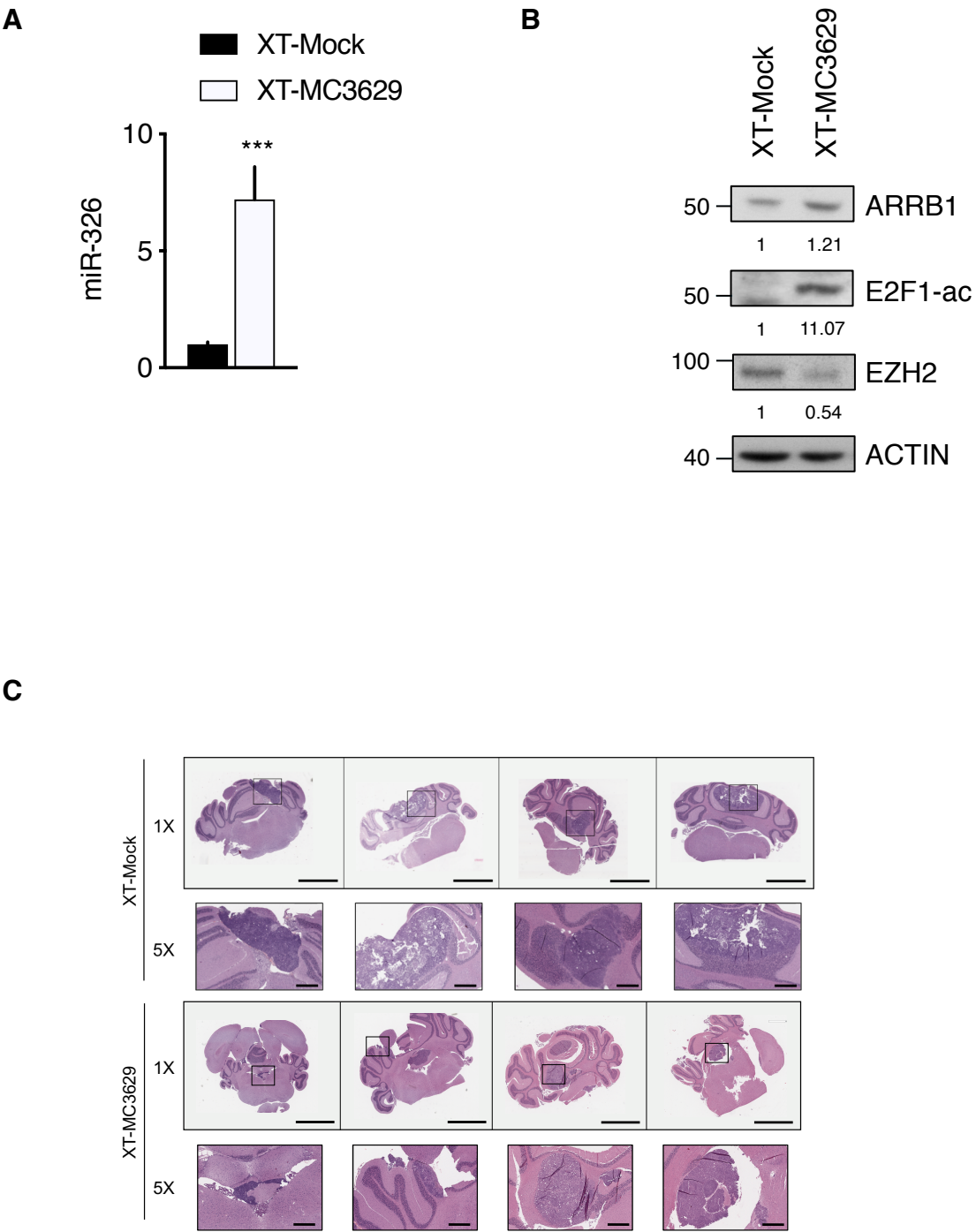

Supplement: Supplementary file 8 — Fig. S8. In vivo pharmacological inhibition of EZH2 in MB CSCs. [file MOL2-15-523-s008.pdf]

Supplementary Figure 9. Ectopic miR-326 and ARRB1 expression inhibits MB cell growth *in vivo*

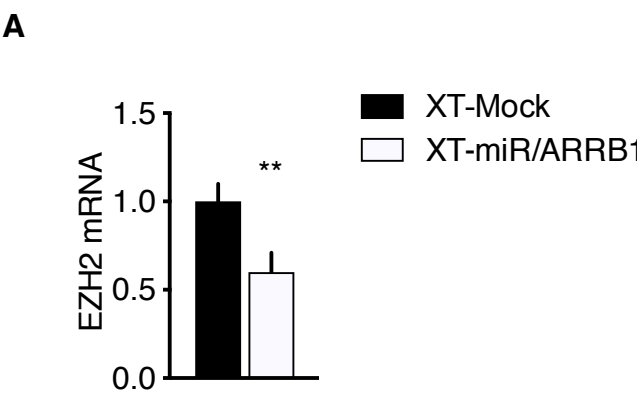

Supplement: Supplementary file 9 — Fig. S9. Ectopic miR‐326 and ARRB1 expression inhibits MB cell growth in vivo. [file MOL2-15-523-s009.pdf]

Supplementary Figure 10. Hematoxylin and eosin staining images of XT *in vivo* experiments

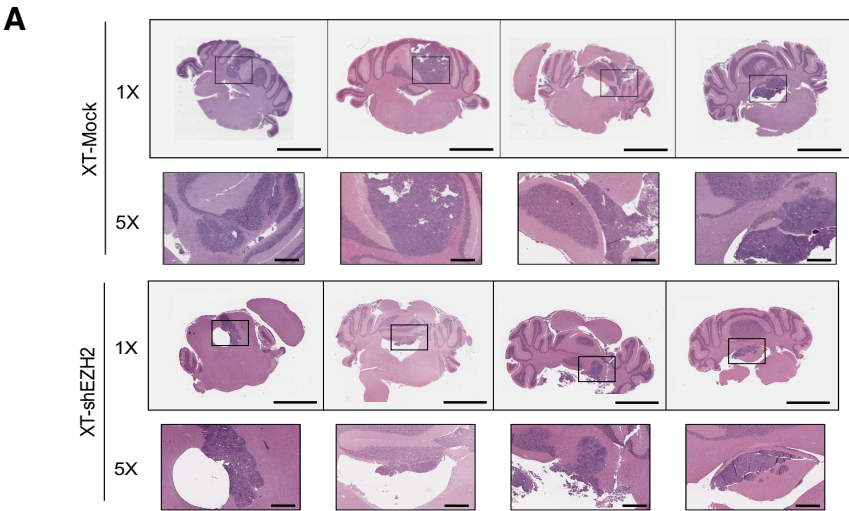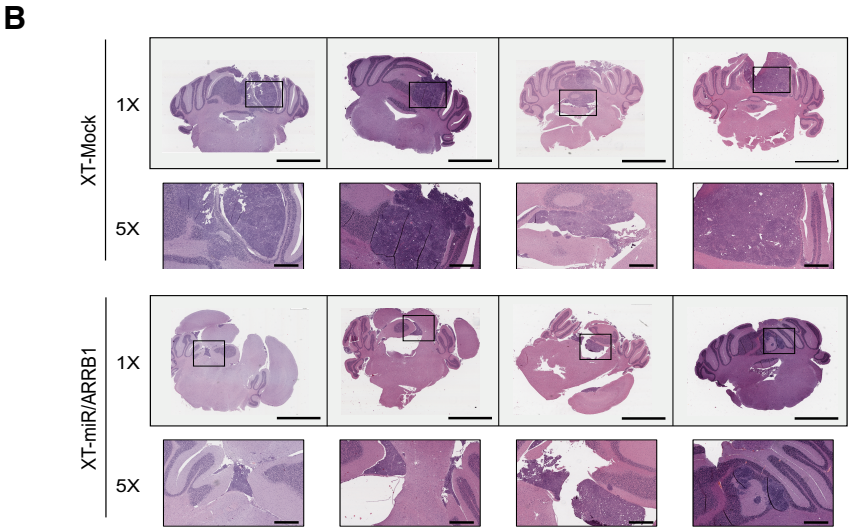

Supplement: Supplementary file 10 — Fig. S10. Hematoxylin and eosin staining images of XT in vivo experiments. [file MOL2-15-523-s010.pdf]
