# Supplementary material for: Downregulation of miR‐326 and its host gene β‐arrestin1 induces pro‐survival activity of E2F1 and promotes medulloblastoma growth
Source: Mol Oncol. 2020 Dec 31;15(2):523–42. doi: 10.1002/1878-0261.12800 (PMC7858128; doi:10.1002/1878-0261.12800)

Supplementary Figure 5. E2F1 expression levels in cohort 2 of MB samples and normal adult cerebella (NAC)

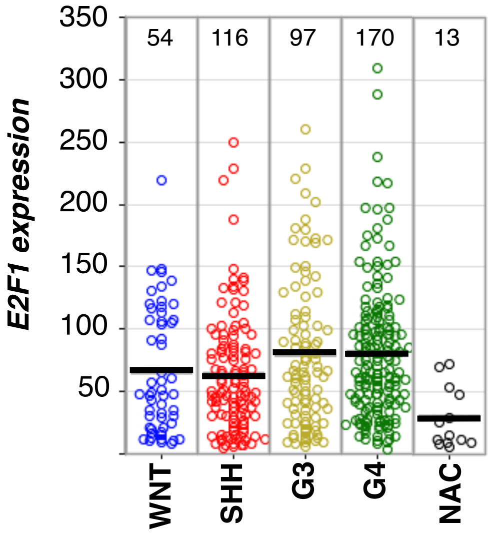

Supplement: Supplementary file 5 — Fig. S5. E2F1 expression levels in cohort 2 of MB samples and normal adult cerebella (NAC). [file MOL2-15-523-s005.pdf]
