# Supplementary material for: Downregulation of miR‐326 and its host gene β‐arrestin1 induces pro‐survival activity of E2F1 and promotes medulloblastoma growth
Source: Mol Oncol. 2020 Dec 31;15(2):523–42. doi: 10.1002/1878-0261.12800 (PMC7858128; doi:10.1002/1878-0261.12800)

Supplementary Figure 7. Characteristics of the orthotopic brain XTs generated in immunocompromised mice using MB CSCs

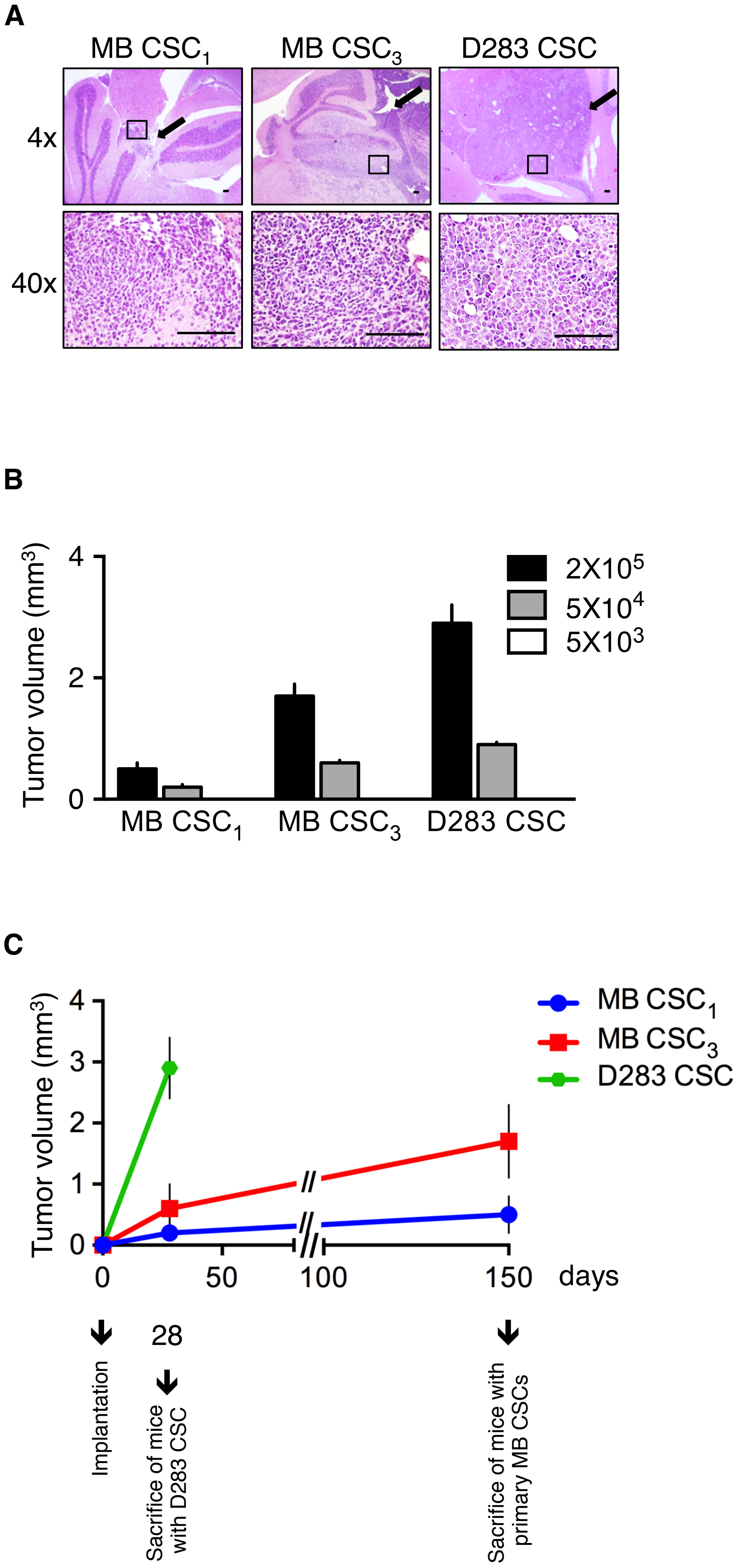

Supplement: Supplementary file 7 — Fig. S7. Characteristics of the orthotopic brain XTs generated in immunocompromised mice using MB CSCs. [file MOL2-15-523-s007.pdf]
