# Supplementary material for: Downregulation of miR‐326 and its host gene β‐arrestin1 induces pro‐survival activity of E2F1 and promotes medulloblastoma growth
Source: Mol Oncol. 2020 Dec 31;15(2):523–42. doi: 10.1002/1878-0261.12800 (PMC7858128; doi:10.1002/1878-0261.12800)
Supplement: Supplementary file 11 — Table S1. Characteristics of cohort 1 tumors. [file MOL2-15-523-s011.pdf]

Supplementary Table 1. Characteristics of cohort 1 tumors

No.	Case ID no.	Histotype*	Molecular Subgroup	Age group†
1	m3	LCA	Group 4	adult
2	m5	C	SHH	adult
3	m6	C	SHH	adult
4	m9	C	Group 3	child
5	m11	D	SHH	adult
6	m13	LCA	SHH	infant
7	m14	C	SHH	child
8	m15	C	Group 4	child
9	m16	C	SHH	infant
10	m17	C	SHH	infant
11	m18	C	Group 3	child
12	m20	C	Group 4	child
13	m21	C	Group 4	child
14	m22	C	Group 4	child
15	m24	LCA	Group 3	infant
16	m25	LCA	SHH	child
17	m26	D	Group 3	child
18	m27	D	SHH	infant
19	m28	C	Group 3	child
20	m29	C	WNT	child
21	m30	C	SHH	child
22	m31	C	Group 3	child
23	m32	C	Group 3	infant
24	m33	D	SHH	infant
25	m34	C	Group 3	child
26	m35	LCA	Group 4	child
27	m36	C	SHH	infant
28	m37	D	SHH	infant
29	m39	LCA	Group 3	child
30	m40	C	SHH	infant
31	m41	C	Group 3	child
32	m42	D	SHH	child
33	m43	C	SHH	infant
34	m44	LCA	SHH	child
35	m45	C	Group 4	child
36	m46	C	Group 3	child
37	m47	LCA	Group 3	child
38	m49	C	SHH	infant
39	m50	C	Group 4	child
40	m51	D	SHH	infant
41	m53	D	SHH	infant
42	m54	LCA	Group 3	infant
43	m57	D	SHH	infant

No.	Case ID no.	Histotype	Molecular Subgroup	Age group†
44	m59	D	Group 4	child
45	m61	LCA	Group 3	child
46	m62	C	SHH	child
47	m63	C	WNT	child
48	m65	LCA	Group 3	infant
49	m67	C	Group 4	child
50	m68	C	Group 3	infant
51	m71	LCA	SHH	child
52	m73	LCA	Group 4	child
53	m75	C	WNT	child
54	m77	LCA	Group 3	child
55	m79	C	Group 3	child
56	m80	C	WNT	child
57	m81	C	Group 4	child
58	m82	C	WNT	child
59	m84	LCA	Group 3	child
60	m85	C	Group 3	infant
61	m86	C	Group 4	child
62	m87	C	WNT	child
63	m88	C	Group 4	child
64	m89	LCA	Group 3	child
65	m90	C	Group 3	infant
66	m91	D	SHH	infant
67	m92	D	SHH	child
68	m93	C	Group 4	child
69	m94	C	WNT	child
70	m95	C	WNT	child
71	m96	LCA	Group 3	child
72	m97	D	SHH	child
73	m101	C	Group 4	child
74	m102	C	SHH	infant
75	m103	C	SHH	infant
76	m105	C	Group 3	child
77	m106	C	Group 4	child
78	m108	LCA	Group 3	child
79	m109	C	Group 4	child
80	m111	LCA	Group 3	child
81	m112	C	Group 3	child
82	m113	C	WNT	child
83	m118	C	WNT	child
84	m120	LCA	Group 3	child

***** C, classic; D,desmoplastic; LCA, large cell/ anaplastic;
† Infant (< 3 y); Child (3-17 y); Adult (>17 y)
