# Supplementary material for: Downregulation of miR‐326 and its host gene β‐arrestin1 induces pro‐survival activity of E2F1 and promotes medulloblastoma growth
Source: Mol Oncol. 2020 Dec 31;15(2):523–42. doi: 10.1002/1878-0261.12800 (PMC7858128; doi:10.1002/1878-0261.12800)
Supplement: Supplementary file 12 — Table S2. List of primers used in Chromatin immunoprecipitation experiments. [file MOL2-15-523-s012.pdf]

Supplementary Table 2. List of primers used in Chromatin immunoprecipitation experiments

**QPCR Primers**	**Sequences**
human ARRB1 Fw:	5'-ACACCCAGCATTGGCCGGCAGCCGCA-3'
human ARRB1 Rev:	5'-CTGTCAGCAAGAATGTGTTGACCGAGGCG-3'
NRE- human ARRB1 exon 5 Fw:	5'-AAGGACCTGTTTGTGGCCAA-3'
NRE- human ARRB1 exon 5 Rev:	5'-CTCGCCCAGCTTCTTGATG-3'
NRE- human ARRB1 exon 8 Fw:	5'-GAATTCTGTGCGTCTGGTCA-3'
NRE- human ARRB1 exon 8 Rev:	5'-GGCTTGTCCGACATGAGG-3'
**PCR Primers**	
human ARRB1 Fw:	5'-ACACCCAGCATTGGCCGGCAGCCGCA-3'
human ARRB1 Rev:	5'-CTGTCAGCAAGAATGTGTTGACCGAGGCG-3'
NRE- human ACTIN Fw:	5'- GCGCTCGGTGAGGATCTTCA-3'
NRE- human ACTIN Rev:	5'- ATGATGATATCGCCGCGCTC-3'
