# Supplementary material for: Downregulation of miR‐326 and its host gene β‐arrestin1 induces pro‐survival activity of E2F1 and promotes medulloblastoma growth
Source: Mol Oncol. 2020 Dec 31;15(2):523–42. doi: 10.1002/1878-0261.12800 (PMC7858128; doi:10.1002/1878-0261.12800)
Supplement: Supplementary file 13 — Appendix S1. Materials and methods. Supplementary Material [file MOL2-15-523-s013.docx]

**Appendix S1- Materials and Methods**

**Western blotting**

The following antibodies were used:

| **Antibody** | **Code** | **Company** |
| --- | --- | --- |
| anti-β-arrestin1 K-16 | sc-8182 | Santa Cruz Biotechnology, Santa Cruz, CA |
| anti-Nanog | REC-RCAB0002P-F | Cosmo Bio Co, Tokyo, Japan |
| anti-CD133 | ab16518 | Abcam, Cambridge, UK |
| anti-mouse βIIItubulin | MAB 1637 | MilliporeSigma - now Merck, Darmstadt, Germany |
| anti-E2F1 KH95 | sc-251 | Santa Cruz Biotechnology |
| anti-E2F1 C- 20 | sc-193 | Santa Cruz Biotechnology |
| anti-E2F1 (acetyl K120/K125) | AP10555SU-N | Acris Antibodies [now Origene Europe], Herford, Germany |
| anti-p300 C-20 | sc-585 | Santa Cruz Biotechnology |
| anti-PARP p85 Fragment | G7342 | Promega, Madison, WI |
| anti-trimethyl-Histone H3 (Lys27) | 07-449 | Merck |
| anti-EZH2 | 4905S | Cell signaling, Danvers, MA |
| anti-Actin I-19 | sc-1616 | Santa Cruz Biotechnology |
| anti-HSP70 | sc-33575 | Santa Cruz Biotechnology |
| anti-GAPDH | ab9484 | Abcam |

**Immunoprecipitation assays**

The following antibodies were used:

| **Antibody** | **Code** | **Company** |
| --- | --- | --- |
| c-Myc 9E10 AC | sc-40AC | Santa Cruz Biotechnology |
| Protein G Plus- Agarose | sc-2002 | Santa Cruz Biotechnology |
| anti-FLAG M2 Affinity Gel | A2220 | Sigma-Aldrich |
| anti-β-arrestin1 | Clone 10 cat. 610550 | BD Biosciences, San Jose, CA |
| anti-E2F1 KH20 | sc-56662 | Santa Cruz Biotechnology |
| anti-HA | sc-7392 | Santa Cruz Biotechnology |
| anti-myc-HRP | Clone 4a6 16-213 | Sigma-Aldrich |
| anti-E2F1 | 3742 | Cell signaling |
| anti-FLAG M2-Peroxidase (HRP) | A8592 | Sigma-Aldrich |
