# Supplementary material for: Downregulation of miR‐326 and its host gene β‐arrestin1 induces pro‐survival activity of E2F1 and promotes medulloblastoma growth
Source: Mol Oncol. 2020 Dec 31;15(2):523–42. doi: 10.1002/1878-0261.12800 (PMC7858128; doi:10.1002/1878-0261.12800)
Supplement: Supplementary file 14 [file MOL2-15-523-s014.docx]

**Supporting Information**

Supplementary Materials and Methods

**Supplementary Table legends**

**Supplementary Table 1. Characteristics of cohort 1 tumors.**

**Supplementary Table 2. List of primers used in Chromatin immunoprecipitation experiments.**

**Supplementary Figure legends**

**Supplementary Figure 1. *miR-326* and *ARRB1* expression in cohort 2 tumors. (A)** Affymetrix microarray analysis of *ARRB1* and miR-sequencing analysis of *miR-326* expression in cohort 2 MBs (n=437 and n=69, respectively) confirm the under-expression of both genes vs. NAC (control) documented in cohort 1 MBs (Fig. 1A, B). Mean expression levels (black horizontal bars) are shown for all MBs subgroups and NAC (controls). Numbers of samples appear at the top of each column. **(B)** Immunohistochemistry (IHC) staining for ARRB1 in sections of NAC and two representative MBs. Magnification: 40X; insets 63X. Scale bar, 100µm.

**Supplementary Figure 2. MB CSCs properties.** MB CSCs were derived from six cohort 1 tumors (CSCs_1-6_) and from D283 cells and characterized as described in Methods. **(A)** Phase-contrast microscopy: Representative images of oncosphere formation observed when MB cells were grown in stem-cell medium (MB CSC) and morphological changes observed when oncosphere cells were transferred to differentiation medium (MB CSC-DFM). Scale bar, 20µm. (**B-C**) Representative immunoblots comparing expression of stem-cell markers (NANOG and CD133) in **(B)** MB CSCs and corresponding BTCs and in **(C)** MB CSCs and MB CSC-DFM. **(D)** Expression of differentiation markers (neuronal: βIIItubulin; glial: GFAP) in MB CSC and MB CSC-DFM: Representative immunofluorescence findings (red: βIIItubulin or GFAP; blue: Hoechst dye. Scale bar, 5 μm) (left) and histogram showing mean+SD percentages of cells expressing each marker under the two conditions (right) (βIIItubulin *p*<0.0001, GFAP *p*<0.0001). Statistics: Two-way ANOVA, *****p*<0.0001. **(E)** Representative immunoblots comparing expression of stem-cell (NANOG and CD133) and neuronal differentiation (βIIItubulin) markers in D283 CSC and D283 CSC-DFM_._ **(F)** Limiting dilution assay of MB CSC clonogenicity (representative findings). Plating densities (cells / well) are indicated on x axes (ranges: 1-500 and 1-100 cells per well for primary and secondary oncosphere formation assays, respectively).

**Supplementary Figure 3. EZH2 mRNA levels in MB samples and normal adult cerebella (NAC).**

**(A)** EZH2 mRNA levels in cohort 1 of MB samples and normal adult cerebella NAC (control) (vs. NAC: WNT *p*=0.009, SHH *p*=0.0005, G3 *p*<0.0001, G4 *p*<0.0001).

**(B)** EZH2 mRNA levels in cohort 2 of MB samples and normal adult cerebella (NAC).

**Supplementary Figure 4. Bivalency signs in MB CSCs miR-326/ARRB1 regulatory region**

ChIP experiments showing (**A**) the H3K4me3/H3K27me3 ratio and (**B**) EZH2 occupancy at the *miR-326/ARRB1* regulatory region before and after transfer of MB CSC to DFM. Data represent means ± SD from 3 independent experiments. Statistics: Wilcoxon signed-rank test for paired data, *****p*<0.0001 vs controls.

**Supplementary Figure 5. E2F1 expression levels in cohort 2 of MB samples and normal adult cerebella (NAC).** Mean expression levels (black horizontal bars) are shown for all four molecular subgroups of MBs (n=437) and NAC (controls n=13). Numbers of samples tested are indicated above columns.

**Supplementary Figure 6. ARRB1 modulates E2F1 acetylation in granule cell precursors (GCPs).** GCPs were treated with Sonic Hedgehog ligand (SHH) and total cell extracts were incubated with anti-ARRB1 antibody (IP), or IgG (negative control) and immunoprecipitated with protein A-coupled beads. Immunoblotting (IB) of the ARRB1 precipitate revealed the co-presence of E2F1 and E2F1-ac, using anti-ARRB1, anti-E2F1 and anti-acetylated-E2F1 (E2F1-ac). One% of the immunoprecipitated cell lysates (INPUT) was immunoblotted with anti-ARRB1, anti-E2F1, anti-acetylated-E2F1 (E2F1-ac) and anti-ACTIN (loading control).

**Supplementary Figure 7. Characteristics of the orthotopic brain XTs generated in immunocompromised mice using MB CSCs.** (**A**) Representative images of H&E-stained, largest-diameter sections of XTs generated with primary MB CSCs (lines 1 and 3) and D283 CSC. Arrows indicate the tumor masses. Scale bar, 100 µm. (**B**) Mean volumes of the three XT lines at animal sacrifice (post-implantation day 90 for XTs generated with primary MB CSCs, post-implantation day 28 for those generated with D283 CSC). Bars represent mean ± SD of four independent experiments. (**C**) XT volumes recorded in the three groups of orthotopic MB CSC transplantation experiments (2x10^5^ cells).

**Supplementary Figure 8. *In vivo* pharmacological inhibition of EZH2 in MB CSCs.** Xenograft tumors (XTs) generated in immunocompromised mice using D283 CSC and treated with EZH2 inhibitor (XT-MC3629) or vehicle (XT-Mock) for 21 days, starting from day 7. XT-MC3629 and XT-Mock expression of: (**A**) miR-326 *p*=0.0001 and (**B**) ARRB1, E2F1-ac and EZH2 protein levels (ACTIN: loading control). (**C**) Hematoxylin and eosin staining images at their largest diameter of orthotopic XTs generated with D283 CSCs and treated with EZH2 inhibitor (XT-MC3629) or vehicle (XT-Mock). For every experimental group, 4 mice were sacrificed at 28 days post implant and processed for immunohistochemistry as described in Materials and methods section. Sections were scanned using Aperio Imagescope (Leica Biosystems). Images of the whole sections were taken at 1X (upper picture for each panel, scale bar 3 mm) and the detail of the tumor mass at 5X (below each whole section, scale bar 500 µm). Data represent means ± SD from 8 independent experiments. Statistics: Wilcoxon signed-rank test for paired data. **p*<0.05*; **p*<0.01*; ***p*<0.001*; ****p*<0.0001 vs. indicated controls.

**Supplementary Figure 9. Ectopic miR-326 and ARRB1 expression inhibits MB cell growth *in vivo*.** Orthotopic XTs were generated in immunocompromised mice by injection of D283 CSCs transduced with separate vectors overexpressing *miR-326* and *ARRB1* (XT-miR/ARRB1) or empty vector (XT-Mock, controls). XTs were assayed for EZH2 levels *p*=0.0017. Data represent means ± SD from 8 independent experiments. Statistics: Wilcoxon signed-rank test for paired data. **p*<0.05*; **p*<0.01*; ***p*<0.001*; ****p*<0.0001 vs. indicated controls.

**Supplementary Figure 10. Hematoxylin and eosin staining images of XT *in vivo* experiments.** Hematoxylin and eosin staining of orthotopic XTs generated in immunocompromised mice by injection of D283 CSCs at their largest diameter. For every experimental group, 4 mice were sacrificed at 28 days post implant and processed for immunohistochemistry as described in Materials and methods section. Sections were scanned using Aperio Imagescope (Leica Biosystems). Images of the whole sections were taken at 1X (upper picture for each panel, scale bar 3 mm) and the detail of the tumor mass at 5X (below each whole section, scale bar 500 µm). (A) Xenograft tumors (XTs) of D283 CSC transduced with lentiviral shEZH2 (XT-shEZH2) or shScramble (XT-Mock). (B) XTs of D283 CSCs transduced with separate vectors overexpressing miR-326 and ARRB1 (XT-miR/ARRB1) or empty vector (XT-Mock).
